# Kaempferol Mitigates *Pseudomonas aeruginosa*-Induced Acute Lung Inflammation Through Suppressing GSK3β/JNK/c-Jun Signaling Pathway and NF-κB Activation

**DOI:** 10.3390/ph18030322

**Published:** 2025-02-25

**Authors:** Jue Wang, Linlin Zhang, Lu Fu, Zheng Pang

**Affiliations:** 1 Innovative Institute of Chinese Medicine and Pharmacy, Shandong University of Traditional Chinese Medicine, Jinan 250355, China; wangjue2299@163.com; 2 Institute of Pharmacy, Shandong University of Traditional Chinese Medicine, Jinan 250355, China; linlin66210@outlook.com; 3 School of Medicine, Shandong University of Traditional Chinese Medicine, Jinan 250355, China; 18810550962@163.com

**Keywords:** kaempferol, *Pseudomonas aeruginosa*, acute pulmonary inflammation, macrophage, GSK3β, JNK/c-Jun, NF-κB

## Abstract

**Background: ***Pseudomonas aeruginosa*, one of the common bacterial pathogens causing nosocomial pneumonia, is characterized as highly pathogenic and multidrug-resistant. Kaempferol (KP), a natural flavonoid, has been shown to exhibit effectiveness in treating infection-induced lung injury. **Methods:** We applied network pharmacology to explore the underlying mechanisms of KP in treating *P. aeruginosa* pneumonia and further validated them through a mouse model of acute bacterial lung infection and an in vitro macrophage infection model. **Results:** The in vivo studies demonstrated that treatment with KP suppressed the production of proinflammatory cytokines, including TNF, IL-1β, IL-6, and MIP-2, and attenuated the neutrophil infiltration and lesions in lungs, leading to an increased survival rate of mice. Further studies revealed that KP treatment enhanced the phosphorylation of GSK3β at Ser9 and diminished the phosphorylation of JNK, c-Jun, and NF-κB p65 in lungs in comparison to the mice without drug treatment. Consistently, the in vitro studies showed that pretreatment with KP reduced the activation of GSK3β, JNK, c-Jun, and NF-κB p65 and decreased the levels of the proinflammatory cytokines in macrophages during *P. aeruginosa* infection. **Conclusions:** KP reduced the production of proinflammatory cytokines by inhibiting GSK3β/JNK/c-Jun signaling pathways and NF-κB activation, which effectively mitigated the *P. aeruginosa*-induced acute lung inflammation and injury, and elevated the survival rates of mice.

## 1. Introduction

*Pseudomonas aeruginosa*, an opportunistic pathogen, tends to cause serious infections in immunocompromised patients. It has become one of the most predominant pathogens causing hospital-acquired infections globally, accounting for 23% of intensive care unit-acquired infections and 26% of ventilator-associated pneumonia cases [1]. The multiple virulence factors of *P. aeruginosa* facilitate its colonization of the respiratory tract and induce host cells to produce inflammatory mediators, which is accompanied by inflammatory cell infiltration, increased alveolar-capillary permeability, and lung epithelial barrier disruption [2,3]. Furthermore, this bacterium can interfere with intracellular signaling pathways and disrupt cell membrane integrity by injecting virulence effectors into the cells and secreting enzymes and toxins that disrupt cell junctions, leading to apoptosis and necrosis [1]. In addition, the Toll-like receptors (TLRs) of the host immune cells recognize the pathogen-associated molecular patterns (PAMPs) of *P. aeruginosa*, initiating multiple signaling pathways that promote the release of inflammatory mediators, which in turn attract inflammatory cells and further upregulate the inflammation level in lungs [4]. Currently, antibiotic therapy remains the major tool for treating *P. aeruginosa* pneumonia [5,6]. However, the antibiotic resistance of *P. aeruginosa* is a severe issue, and it shows increasing resistance to many commonly used antibiotics [7]. It has been found that *P. aeruginosa* is capable of forming biofilms, which not only physically shield the bacteria from antibiotic penetration but also enable the bacteria to develop resistance mutations in this state, resulting in a major therapeutic challenge [8]. In addition, the efflux pumps of *P. aeruginosa* reduce antibiotic susceptibility by expelling the antibiotics out of bacterial cells, leading to the development of multidrug resistance, and it promotes the formation of biofilm, which makes the bacteria more difficult to eradicate [9]. Antibiotic abuse has resulted in the emergence of multidrug-resistant *P. aeruginosa* strains. Statistically, 23–46% of *P. aeruginosa* infections that cause pneumonia in intensive care units are multidrug-resistant [10]. Consequently, there is an urgent need to find new alternative agents to treat the infections caused by multidrug-resistant *P. aeruginosa* [7]. Natural bioactive compounds have been shown to have excellent pharmacological activities both in preventing the infection of *P. aeruginosa* and modulating the host immune response [11]. Additionally, natural flavonoid compounds have been shown to treat acute lung injury by activating antioxidant pathways and inhibiting inflammatory pathways [12].

Kaempferol (KP), a dietary flavonoid widely distributed in the plant kingdom, exerts anti-inflammatory, antioxidant, antibacterial, anticancer, neuroprotective, and hepatoprotective effects [13,14,15,16]. Previous studies have reported that KP can reduce intestinal inflammation [17], airway inflammation [18], and neuroinflammation [19,20]; ameliorate inflammatory bone disease [21]; and protect blood vessels from inflammatory injury [22], demonstrating the broad anti-inflammatory effects of KP. In particular, KP was identified to attenuate the lipopolysaccharide (LPS)- or virus-induced lung injury by inhibiting MAPK and NF-κB signaling pathways [23,24,25]. Additionally, KP has been found to inhibit a variety of pathogenic bacteria [26,27]. A previous study found that KP reduced the *Staphylococcus aureus*-induced pneumonia by attenuating the production of virulence factors [28]. In addition, many KP-containing herbs have exhibited antimicrobial activity against *P. aeruginosa* [27]. However, the efficacy of KP on *P. aeruginosa*-induced acute pneumonia and the underlying mechanisms have not yet been elucidated.

This research utilized network pharmacology to predict the therapeutic mechanisms of KP in treating P. aeruginosa pneumonia. Glycogen synthase kinase 3 beta (GSK3β) was one of the key targets in the protein–protein interaction (PPI) network, and it has been revealed to exert a crucial function in modulating the activity of inflammation-related proteins [29,30]. Previous studies have shown that the activity of GSK3β was significantly enhanced in the P. aeruginosa-induced keratitis and the inhibition of its activity could downregulate the corneal inflammation [31]. The alteration of GSK3β activity has been found to act on its downstream targets, JNK, c-Jun, and NF-κB, to regulate inflammatory responses [30,32]. Moreover, JNK, c-Jun, and NF-κB p65 were also the key targets identified in the PPI network, and JNK, c-Jun, and NF-κB were identified to regulate the lung inflammation induced by P. aeruginosa infection [33,34,35]. In addition, the data of molecular docking suggested that KP exhibited high binding affinities with the protein targets, including GSK3β, JNK1, and NF-κB p65. Therefore, we hypothesized that KP mitigates P. aeruginosa pneumonia by inhibiting GSK3β/JNK/c-Jun and NF-κB signaling pathways. To validate it, we employed a murine model of P. aeruginosa pneumonia. The results indicated that treatment with KP dampened the P. aeruginosa-induced lung damage by reducing the production of TNF, IL-1β, IL-6, and macrophage inflammatory protein (MIP)-2, and impairing neutrophil infiltration, thus leading to a reduced mortality rate of mice. Moreover, the phosphorylation of GSK3β (Ser9) was increased, whereas the phosphorylation of JNK, c-Jun, and NF-κB p65 was decreased in the lung tissues of the P. aeruginosa-infected mice after KP treatment. Alveolar macrophage is one of the major contributors to the inflammatory cytokine storm in patients with bacterial pneumonia [36]. In addition, macrophages play an important role in host defense against P. aeruginosa infection by recognizing bacterial molecules and activating the inflammatory response [37]. Therefore, we used macrophages to study the in vitro efficacy of KP against P. aeruginosa infection. Similarly, pretreatment with KP resulted in reduced proinflammatory cytokine production; impaired phosphorylation of JNK, c-Jun, and NF-κB p65; and upregulated the phosphorylation of GSK3β at Ser9 in the P. aeruginosa-infected macrophages. Overall, these findings revealed that KP was a potential therapeutic agent for combating acute P. aeruginosa pneumonia, which could alleviate the lung inflammation through suppressing GSK3β/JNK/c-Jun signaling pathway and NF-κB activation.

## 2. Results

### 2.1. Network Pharmacology-Based Prediction of the Mechanisms of KP in the Treatment of P. aeruginosa Pneumonia

**Figure 1:**
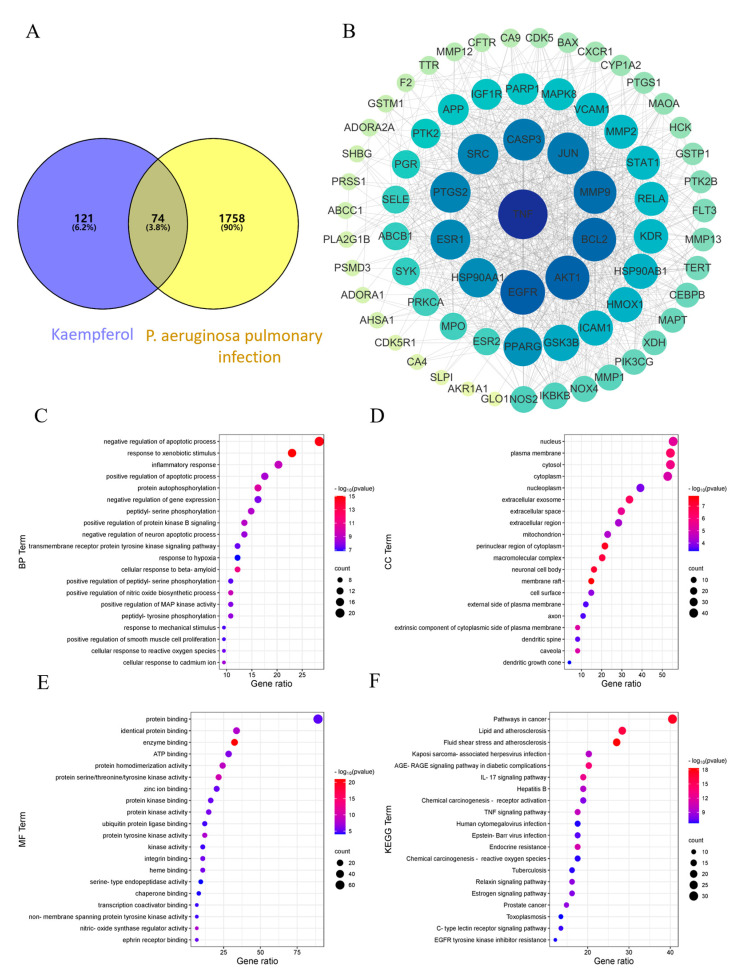
Prediction of the mechanisms of KP in treating *P. aeruginosa* pneumonia by network pharmacology. A total of 121 KP targets and 1758 *P. aeruginosa* pulmonary infection-related targets were collected, and 74 potential targets of KP-*P. aeruginosa* pulmonary infection were obtained by establishing a Venn diagram (**A**). The gray edges in the PPI network represented the potential connections between the targets, while the size and color depth of a node corresponding to the target were positively correlated with the degree of association with other targets (**B**). Gene Ontology (GO) enrichment included biological process (BP) (**C**), cellular component (CC) (**D**), and molecular function (MF) (**E**). Kyoto Encyclopedia of Genes and Genomes (KEGG) enrichment analysis was utilized to obtain the KP-regulated signaling pathways (**F**). Data were displayed as bubble charts. The vertical axis represented the GO or KEGG pathway items, and the horizontal axis represented the number of target genes in each item. The size of the bubble represented the percentage. The colors of the bubbles were correlated with their *p*-values.

Seventy-four overlapping targets were identified between KP and *P. aeruginosa* pulmonary infection, which were regarded as the potential targets of KP in treating *P. aeruginosa* pneumonia (Figure 1A). The interactions at the protein level of the potential targets were obtained by the PPI network (Figure 1B). The top 25 targets in Figure 1B were shown in Table 1 with degree values. The larger size and darker coloration of the target signify a higher degree of centrality, indicating a more important role as a core protein in binding to other proteins. Among the core targets, TNF, EGFR, AKT1, MMP9, JUN, PTGS2, PPARG, GSK3B, ICAM1, HMOX1, RELA, and MAPK8 have been found to play essential roles in the *P. aeruginosa*-induced inflammatory response [31,38,39,40,41,42,43,44]. Moreover, 461 Gene Ontology (GO) terms, including 331 biological process (BP), 48 cellular component (CC), and 82 molecular function (MF) terms were obtained, and the top 20 items were displayed according to their *p*-values (Figure 1C–E). The size of the bubbles in the graph was correlated with the number of targets involved in each term, and the color was correlated with the *p*-value. The predominant processes of BP included the negative regulation of the apoptotic process, the response to xenobiotic stimulus, protein autophosphorylation, inflammatory response, the negative regulation of gene expression, and the response to hypoxia (Figure 1C). The CC clusters predominantly included the nucleus, plasma membrane, cytosol, membrane raft, extracellular exosome, extracellular space, and perinuclear region of the cytoplasm (Figure 1D). The MF clusters mainly involved functions such as protein binding, enzyme binding, protein serine/threonine/tyrosine kinase activity, and protein kinase activity and binding (Figure 1E). These data suggested that KP might regulate the inflammatory response to *P. aeruginosa* infection by influencing the protein binding and activity in the cell membrane, cytoplasm, and nucleus. Furthermore, 123 Kyoto Encyclopedia of Genes and Genomes (KEGG) terms were identified, and the top 20 terms ranked according to the number of involved targets were shown in Figure 1F. The top 20 KEGG enriched pathways sorted by *p*-value were listed in Table 2. In particular, the IL-17, TNF, MAPK, and PI3K-AKT signaling pathways have been found to promote the *P. aeruginosa*-induced lung inflammation [33,34,35,44]. These findings suggested that the regulation of the inflammatory response could be the possible mechanism of KP in treating *P. aeruginosa* pneumonia. 

### 2.2. KP Reduces the Mortality but Has a Limited Effect on Bacterial Clearance in the Lungs of Mice

**Figure 2:**
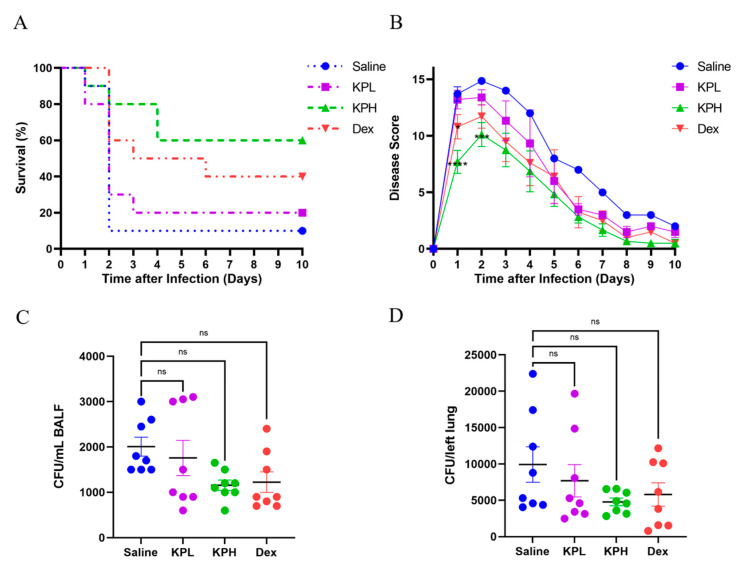
Treatment with KP enhances survival rates but has a limited impact on the bacterial clearance in the lungs during acute *P. aeruginosa* lung infection. The mice from each group were administered 1 × 10^9^ colony-forming unit (CFU)/mouse of PAO1 intranasally for the survival study. The survival rates (**A**) and disease scores (**B**) were recorded daily for 10 days after infection (data are expressed as mean ± standard error of the mean (SEM); *n* = 10; * *p* < 0.05, *** *p* < 0.001, **** *p* < 0.0001). For analyzing bacterial burden, the mice were treated with 5 × 10^8^ CFU of PAO1 per mouse intranasally, and the CFU number in bronchoalveolar lavage fluid (BALF) (**C**) and the lungs (**D**) were analyzed after 24 h of infection (data are expressed as mean ± SEM; *n* = 8; ns: non-significant).

A survival study was conducted to evaluate the therapeutic effects of KP on *P. aeruginosa*-induced acute pneumonia (Figure 2A). The results showed that the survival rates of the mice of kaempferol low-dose (KPL) (20%) and kaempferol high-dose (KPH) (60%) groups were both higher than that of the mice of the Saline group (10%). Moreover, the survival rate of the mice in the KPH group was higher than that in the dexamethasone (Dex) group (40%), demonstrating a better therapeutic effect of KPH than Dex. Consistently, the disease scores of the mice from the KP-treated groups were lower than those of the mice of the Saline group (Figure 2B). These results indicate that KP ameliorated the disease status of the *P. aeruginosa* lung-infected mice and decreased their mortality, suggesting the therapeutic and protective effects of KP on *P. aeruginosa*-induced acute pneumonia. To further explore the in vivo inhibitory effect of KP on *P. aeruginosa*, the bacterial burden in bronchoalveolar lavage fluid (BALF) and lung tissues was examined (Figure 2C,D). The data revealed that the number of bacteria in the BALF and lungs was reduced in the KP-treated groups in comparison to the Saline group but did not attain statistical significance. This indicated that KP exerted a limited effect on bacterial clearance in vivo, and the increased survival rate of the KP-treated mice might be attributed to the alleviated inflammatory response in the lungs.

### 2.3. KP Dampens the P. aeruginosa-Induced Acute Lung Injury and Impairs Neutrophil Recruitment

**Figure 3:**
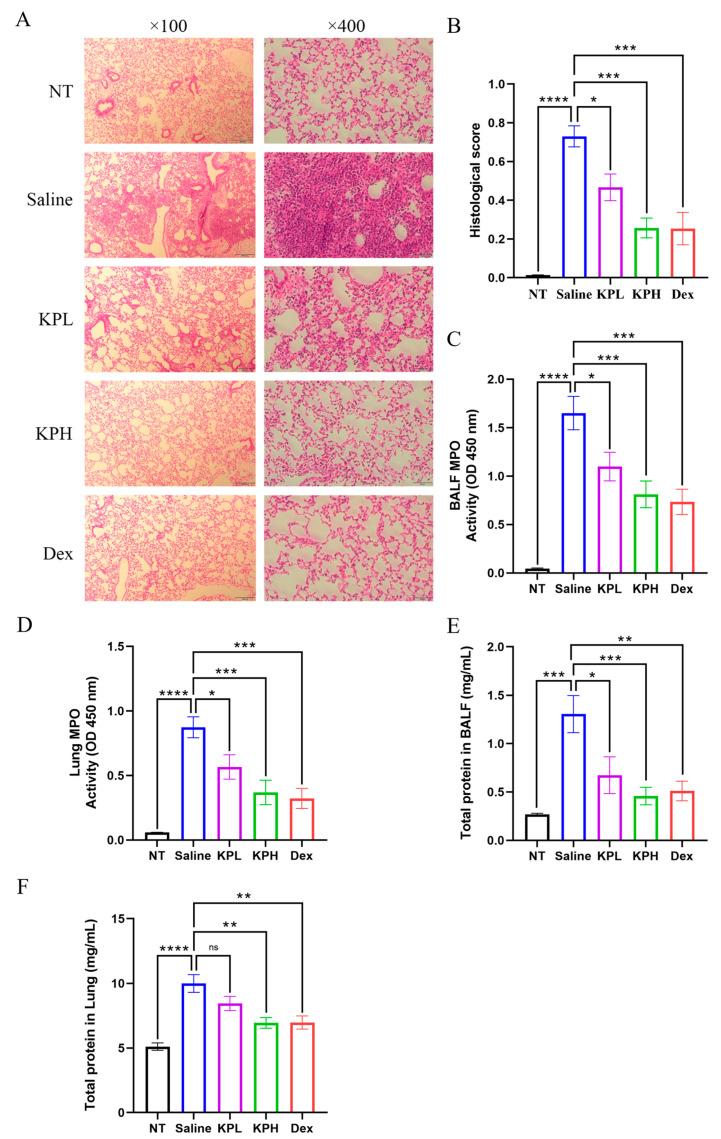
KP ameliorates the *P. aeruginosa*-induced acute lung injury, reduces neutrophil infiltration, and decreases pulmonary vascular permeability. The mice from the Saline, kaempferol low-dose (KPL), kaempferol high-dose (KPH), and dexamethasone (Dex) groups were intranasally administered with 5 × 10^8^ CFU/mouse of PAO1 for 24 h, whereas the mice from the uninfected (NT) group received a mock infection with saline. The post-caval lobes of the lungs were subjected to hematoxylin and eosin (H&E) staining, and imaged at magnifications of 100× and 400× (**A**). The histological score of lung injury was evaluated in each mouse (**B**) (data are expressed as mean ± SEM; *n* = 6; * *p* < 0.05, *** *p* < 0.001, **** *p* < 0.0001). The myeloperoxidase (MPO) activity was examined in the BALF (**C**) and lung (**D**) lysates. The total protein content of BALF (**E**) and left lung (**F**) was detected by bicinchoninic acid (BCA) assay (data are expressed as mean ± SEM; *n* = 8; * *p* < 0.05, ** *p* < 0.01, *** *p* < 0.001, **** *p* < 0.0001, ns: non-significant).

Hematoxylin and eosin (H&E) staining was applied to assess the influence of KP on the *P. aeruginosa*-induced lung injury. The images displayed that the mice in the Saline group had serious lung tissue lesions with obvious alveolar collapse and alveolar septal thickening associated with the infiltration of a large number of inflammatory cells. By contrast, the lesions were significantly attenuated in the KP-treated groups (Figure 3A). Furthermore, the KP-treated group had lower histological scores of lung injury compared with those of the Saline group (Figure 3B). Neutrophil recruitment is one of the important characteristics of acute *P. aeruginosa* pneumonia, and the excessive infiltration of neutrophils can cause severe damage in lung tissue [34]. Myeloperoxidase (MPO), a heme-containing peroxidase predominately produced in neutrophils, is commonly used as a biomarker for quantitatively assessing the number of infiltrated neutrophils. The data showed that the MPO activity was significantly increased in both the lung tissues and BALF of mice upon *P. aeruginosa* lung infection. However, the MPO activity of BALF and lungs was observably decreased in the KP-treated mice in a dose-dependent manner (Figure 3C,D). The excessive inflammatory response in the lungs can lead to a cytokine storm characterized by a significant increase in cytokines, such as TNFα, IL-6, IL-1β, and IFNγ, and increase vascular permeability, which causes blood proteins to leak into the alveoli [45,46]. The total protein concentration of BALF and lung tissues was measured by bicinchoninic acid (BCA) assay, and the results showed that KP treatment was able to dose-dependently reduce the protein concentrations of the BALF and lung tissues in mice after infection (Figure 3E,F). These findings suggested that treatment with KP ameliorated histopathological changes, impaired neutrophil infiltration, and reduced pulmonary vascular permeability during *P. aeruginosa* lung infection.

### 2.4. KP Inhibits the P. aeruginosa-Induced Proinflammatory Cytokine Production in Lungs

**Figure 4:**
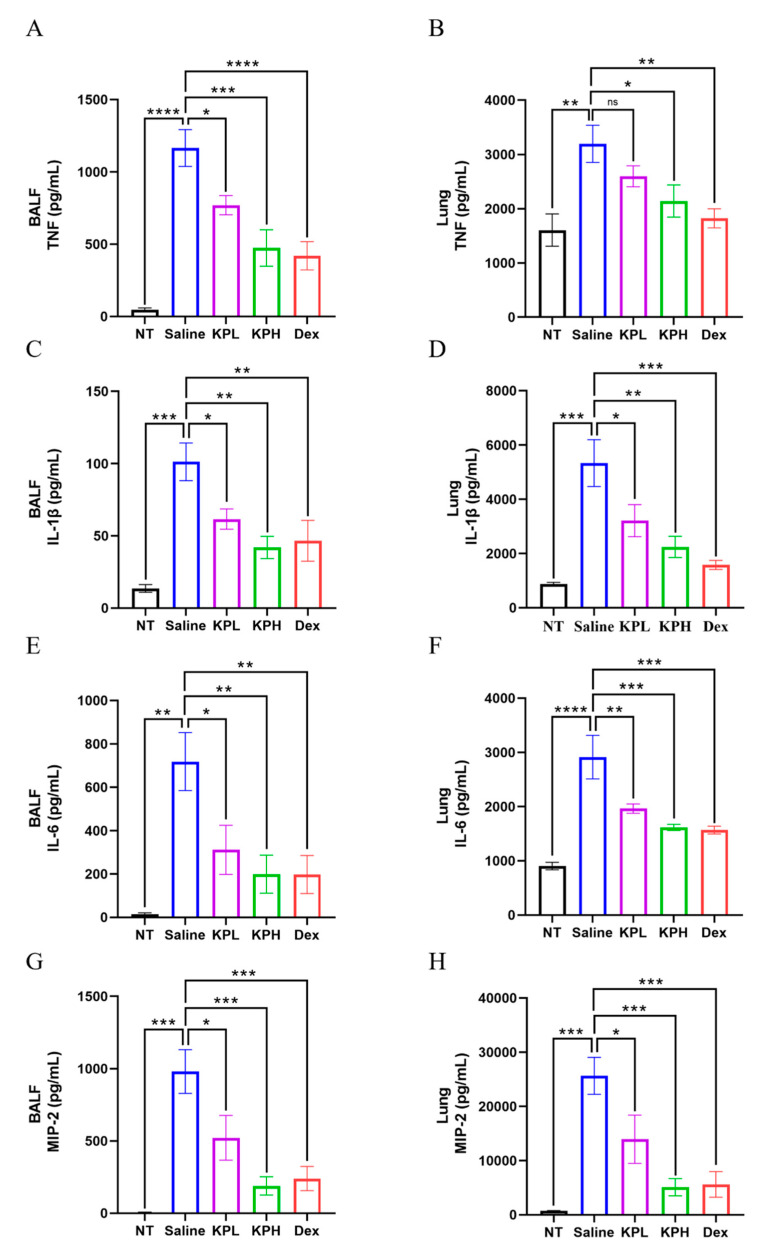
KP inhibits the production of proinflammatory cytokine in the lungs during *P. aeruginosa* acute pulmonary infection. The mice in the Saline, KPL, KPH, and Dex groups were given 5 × 10^8^ CFU of PAO1 per mouse through intranasal infection for 24 h, whereas the mice in the NT group were received a mock infection with saline. The levels of TNF (**A**,**B**), IL-1β (**C**,**D**), IL-6 (**E**,**F**), and macrophage inflammatory protein (MIP)-2 (**G**,**H**) in the BALF and lung tissues of the mice were determined by ELISA (Data are expressed as mean ± SEM; *n* = 8; * *p* < 0.05, ** *p* < 0.01, *** *p* < 0.001, **** *p* < 0.0001, ns: non-significant).

In response to infections, the host body produces proinflammatory cytokines to activate and modulate the immune response. However, the overproduction of cytokines leads to excessive inflammation and damage to tissues [45]. The levels of cytokines, TNF, IL-1β and IL-6, and chemokine MIP-2 in lungs were previously reported to be upregulated after *P. aeruginosa* infection [47]. We examined the levels of TNF, IL-1β, IL-6, and MIP-2 in the BALF and lung tissues of mice by enzyme-linked immunosorbent assay (ELISA) (Figure 4). The results showed that the levels of TNF, IL-1β, IL-6, and MIP-2 were significantly elevated in both the BALF and lung tissues of mice after *P. aeruginosa* lung infection, while KP treatment reduced the levels of these cytokines in a dose-dependent manner. To exclude the confounding factors caused by KP, the effects of KP on cytokine production in uninfected mice were examined, and we found that the levels of TNF, IL-1β, IL-6, and MIP-2 in the lungs of KP groups were not significantly different from those of Saline group (Figure S1). Our data showed that KP exhibited an inhibitory effect on the *P. aeruginosa*-induced pneumonia in vivo.

### 2.5. Binding Activity of KP with GSK3β, JNK1, c-Jun, and NF-κB p65

**Figure 5:**
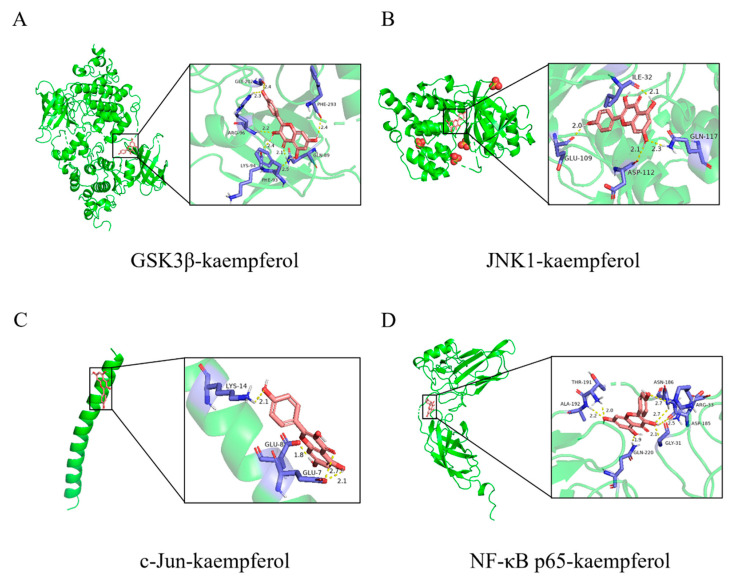
Molecular docking of KP with target proteins. KP was docked with glycogen synthase kinase 3 beta (GSK3β) (**A**), JNK1 (**B**), c-Jun (**C**), and NF-κB p65 (**D**), and the data were visualized using PyMOL 2.4.0.

GSK3β (GSK3B) and its downstream proteins, JNK1 (MAPK8), c-Jun (JUN), and NF-κB p65 (RELA) were identified through network pharmacology screening and molecularly docked with KP to further predict the ability of KP to interact with these targets (Figure 5). Table 3 demonstrated the binding energies of KP to the core target proteins, GSK3β, JNK1, c-Jun, and NF-κB p65. The smaller value of the binding energy indicates that easier intermolecular binding occurs, and it is widely accepted that the high binding affinity between drugs and protein molecules occurs when the binding energy is below −5 kcal/mol [34,35]. The docking results showed a good binding affinity of KP with GSK3β (−5.44 kcal/mol), JNK1 (−5.03 kcal/mol), and NF-κB p65 (−5.56 kcal/mol). This finding implied that KP might regulate the inflammatory response by affecting the binding capacity of these proteins or modulating their activation levels.

### 2.6. KP Suppresses GSK3β/JNK/c-Jun Signaling Pathway and NF-κB Activation In Vivo

**Figure 6:**
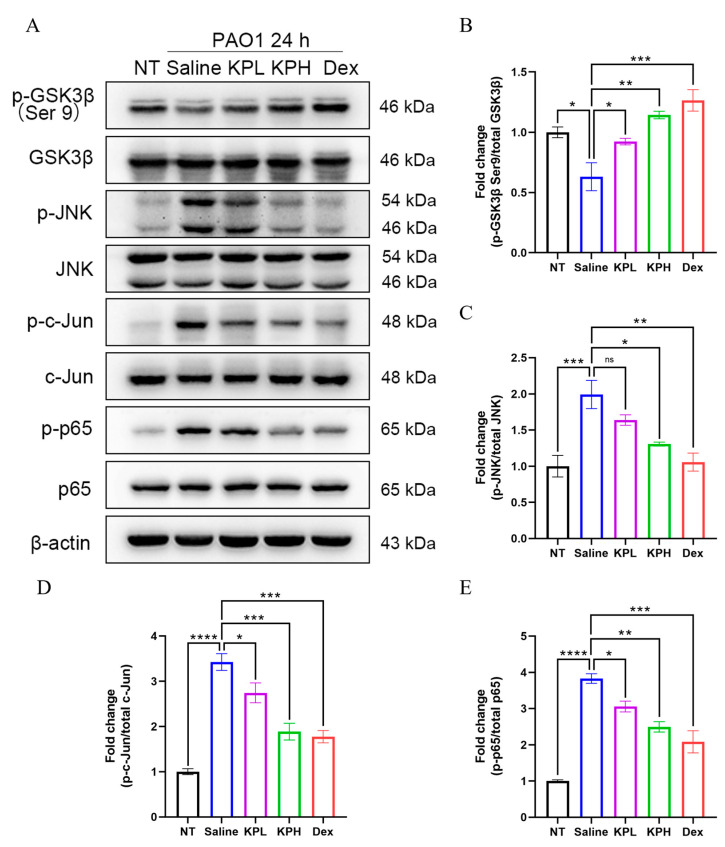
KP inhibits the activation of GSK3β, JNK, c-Jun, and NF-κB p65 in the lungs of mice infected with *P. aeruginosa*. The phosphorylation of GSK3β (Ser9), JNK, c-Jun, and NF-κB p65 in the lung lysates of the mice from the NT, Saline, KPL, KPH, and Dex groups was examined by Western blot analysis (**A**). Densitometry analysis of the phosphorylated GSK3β (Ser9) (**B**), JNK (**C**), c-Jun (**D**), and NF-κB (**E**) was normalized to their total proteins (data are expressed as mean ± SEM; *n* = 3; * *p* < 0.05, ** *p* < 0.01, *** *p* < 0.001, **** *p* < 0.0001, ns: non-significant). β-actin was applied as the loading control.

GSK3β is active under basal conditions and inactivated by phosphorylation at Ser9 [30]. The PAMPs from pathogens are recognized by TLRs and increase the activity of GSK3β [48,49]. Furthermore, the increased activity of GSK3β aggravates inflammatory response, whereas GSK3β inhibition has been proven to alleviate lung injury [29,50,51]. GSK3β activates JNK by phosphorylating MEKK1 [52] and increases IκB degradation and NF-κB activation by regulating IκB kinase [53]. In addition, previous studies showed that GSK3β can also directly phosphorylate NF-κB p65 [54]. To assess the effect of KP on the GSK3β/JNK/c-Jun and NF-κB signaling pathways in vivo, the expression or phosphorylation levels of GSK3β, JNK, c-Jun, and NF-κB p65 in the lungs were examined (Figure 6 and Figure S2). The results showed that neither *P. aeruginosa* infection nor drug treatment significantly affected the mRNA transcription and protein expression of GSK3β, JNK, c-Jun, and NF-κB p65. Upon *P. aeruginosa* lung infection, the phosphorylation levels of JNK, c-Jun and NF-κB p65 were significantly increased, while the phosphorylation of GSK3β at Ser9 was decreased. Furthermore, the phosphorylation of JNK, c-Jun and NF-κB p65 was downregulated, whereas the phosphorylation level of GSK3β at the Ser9 was significantly increased in the KP-treated groups compared with the Saline group. These results suggested that KP inhibited the GSK3β/JNK/c-Jun signaling pathway and NF-κB activation in treating the *P. aeruginosa*-induced acute pneumonia.

### 2.7. KP Reduces the Expression of Proinflammatory Cytokines in Macrophages During P. aeruginosa Infection

**Figure 7:**
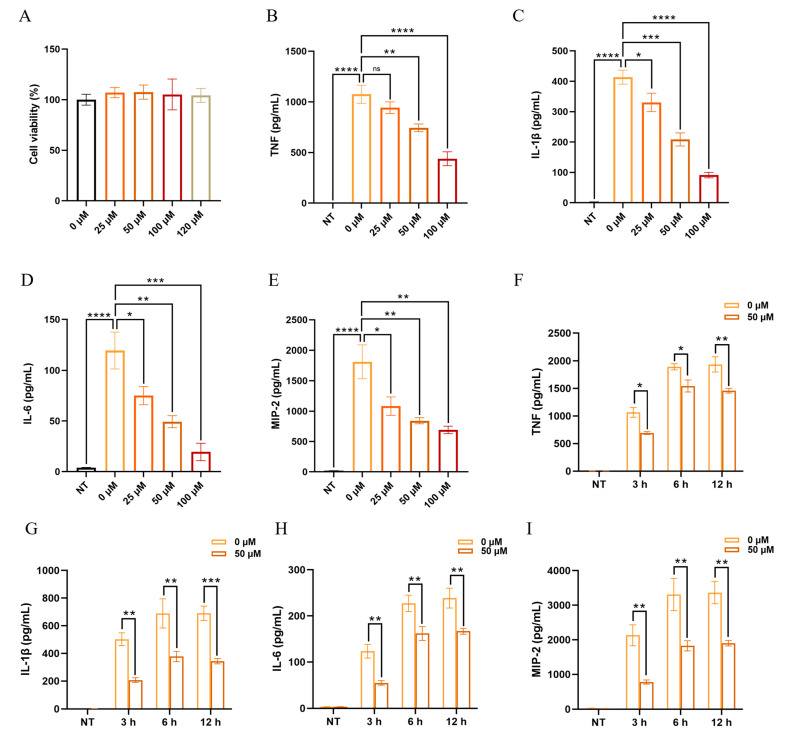
Pretreatment with KP reduces the proinflammatory cytokine production in macrophages upon *P. aeruginosa* infection. The cell viability of KP- (25, 50, 100, and 120 μM) or mock-treated mouse bone-marrow-derived macrophages (BMDMs) was detected with a CCK-8 kit (**A**) (Data are expressed as mean ± SEM; *n* = 4). BMDMs were pretreated with KP (0, 25, 50, and 100 μM) for 1 h and then infected with PAO1 or mock-infected (NT) for 3 h. The levels of TNF (**B**), IL-1β (**C**), IL-6 (**D**), and MIP-2 (**E**) in the cell-free supernatants were determined by enzyme-linked immunosorbent assay (ELISA). For time-course experiments, BMDMs were pretreated with 50 μM KP or PBS (0 μM) for 1 h and then infected with PAO1 for 3 h, 6 h and 12 h. The levels of TNF (**F**), IL-1β (**G**), IL-6 (**H**), and MIP-2 (**I**) were determined in cell-free supernatants. (Data are expressed as mean ± SEM; *n* = 3; * *p* < 0.05, ** *p* < 0.01, *** *p* < 0.001, **** *p* < 0.0001, ns: non-significant).

Macrophages, the sentinel cells in the lungs, regulate the inflammatory response by recognizing bacteria and secreting inflammatory mediators [37]. Mouse bone marrow-derived macrophages (BMDMs) were used for further in vitro experiments. No significant cytotoxic effect was found across a range of KP concentrations (25, 50, 100, and 120 μM) compared to the blank control group (Figure 7A). The levels of proinflammatory cytokines including TNF, IL-1β, IL-6, and MIP-2 were measured in the cell-free supernatants of the *P. aeruginosa*-infected BMDMs at 3 h post-infection (Figure 7B–E). The results showed that the *P. aeruginosa* significantly induced the production of these cytokines, which were decreased dose-dependently in the cells pretreated with KP at different concentrations (25, 50, and 100 μM). In addition, we conducted a time-course experiment to assess the duration of the effect of KP on cytokine production. The results showed that KP exhibited an inhibitory effect on the production of TNF, IL-1β, IL-6, and MIP-2 in the *P. aeruginosa*-infected BMDMs at 3, 6, and 12 h (Figure 7F–I). It suggested that KP diminished the expression of proinflammatory cytokines in BMDMs during *P. aeruginosa* infection in vitro.

### 2.8. KP Inhibits the Activation of GSK3β, JNK, c-Jun, and NF-κB in Macrophages During P. aeruginosa Infection

**Figure 8:**
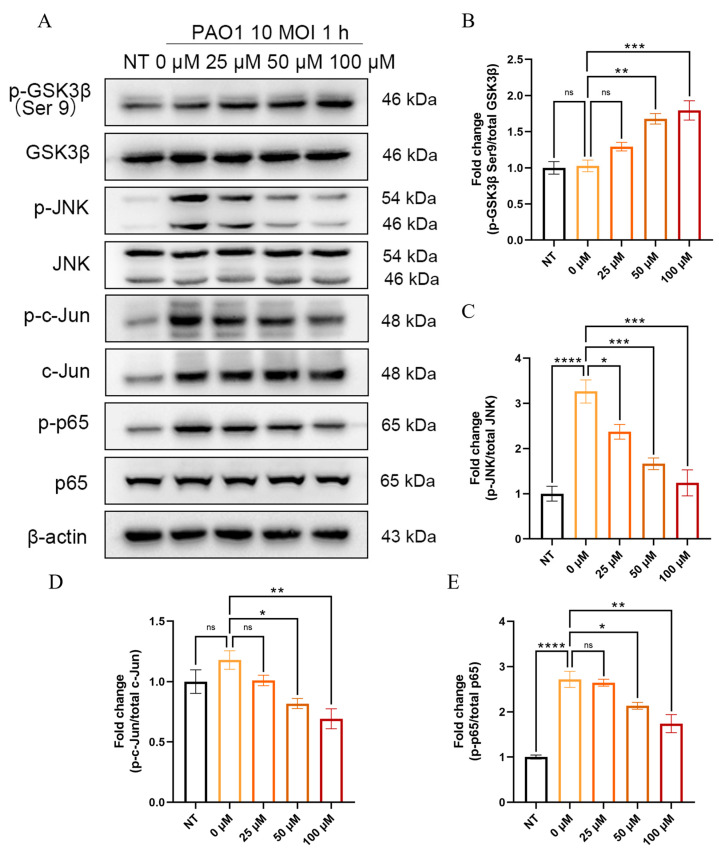
KP diminishes the activation of GSK3β, JNK, c-Jun, and NF-κB p65 in macrophages during *P. aeruginosa* infection. BMDMs were pretreated with KP (0, 25, 50, and 100 μM) for 1 h and then infected with PAO1 or mock-infected (NT) for 1 h. The phosphorylation of GSK3β (Ser9), JNK, c-Jun, and NF-κB p65 in BMDMs was examined by Western blot analysis (**A**). The densitometry analysis of the phosphorylated GSK3β (**B**), JNK (**C**), c-Jun (**D**), and NF-κB p65 (**E**) was normalized to their total proteins (data are expressed as mean ± SEM; *n* = 3; * *p* < 0.05, ** *p* < 0.01, *** *p* < 0.001, **** *p* < 0.0001, ns: non-significant). β-actin was applied as the loading control.

To investigate the impact of KP on GSK3β/JNK/c-Jun and NF-κB signaling pathways in macrophages, the expression and phosphorylation levels of GSK3β, JNK, c-Jun, and NF-κB p65 were analyzed in the KP-pretreated BMDMs upon *P. aeruginosa* infection (Figure 8 and Figure S3). The results showed that *P. aeruginosa* infection did not significantly affect the mRNA transcription and protein expression of GSK3β, JNK, and NF-κB p65 but significantly increased the activation of these proteins. Interestingly, *P. aeruginosa* infection elevated the mRNA and protein expression levels of c-Jun in BMDMs and increased its phosphorylation as well. Furthermore, KP dose-dependently increased the phosphorylation of GSK3β at Ser9 and decreased the phosphorylation of JNK, c-Jun, and NF-κB p65. Those results were consistent with the in vivo results, indicating that KP could attenuate GSK3β/JNK/c-Jun signaling pathway and NF-κB activation in BMDMs during *P. aeruginosa* infection.

## 3. Discussion

*Pseudomonas aeruginosa* commonly causes nosocomial infections, and the mortality of ventilator-associated pneumonia induced by *P. aeruginosa* infection is 32–42.8% [1]. The lung infection caused by this bacterium activates many inflammatory pathways, which can cause severe lung damage [2]. Furthermore, *P. aeruginosa* utilizes endogenous and acquired resistance mechanisms against most antibiotics, which makes the treatment much more difficult [7]. There is an urgent need to develop novel antibiotics and find alternative therapeutic strategies.

Natural bioactive compounds have displayed promising potential in combating *P. aeruginosa* infections and can be used as alternative or complementary drugs to conventional antibiotics [11]. Flavonoids are plant secondary metabolites that have been found to possess antimicrobial and anti-inflammatory activities [55,56]. In addition, some flavonoids have shown considerable efficacy in treating the *P. aeruginosa*-induced lung injury [35,57,58]. For example, quercetin effectively reduced the expression of the *P. aeruginosa*-induced proinflammatory cytokines by repressing NF-κB pathway in H292 cells [58], and it has been demonstrated to mitigate *P. aeruginosa* pneumonia in mice by modulating the PI3K/AKT/NF-κB signaling pathway [35]. Baicalin was identified to alleviate acute pneumonia induced by multidrug-resistant *P. aeruginosa* by inhibiting the TLR4/NF-κB pathway [57]. KP belongs to the flavonoid family and is a safe and effective natural dietary compound with anti-inflammatory and antimicrobial properties [13]. The effects of KP on treating the *P. aeruginosa*-induced acute pneumonia and the underlying mechanisms are unknown.

This research first utilized network pharmacology to study the underlying mechanisms of KP in treating *P. aeruginosa* pneumonia. The results showed that TNF was located in the center of the PPI network, and JUN, GSK3B, RELA, and MAPK8 were listed in the top 25 core targets. KP was previously reported to function as an inhibitor of GSK3β [59], which is regarded as a central regulator of the immune response to bacterial infection and is involved in many bacterial-induced inflammatory diseases by regulating the expression of inflammatory mediators [29,48]. Moreover, GSK3β is inhibited through phosphorylation at Ser9, and the absence or inhibition of this protein has been found to dampen the activation of JNK and NF-κB, leading to decreased expression of proinflammatory cytokines [30,32]. JNK is a member of the MAPK family that can phosphorylate and activate c-Jun, which forms a dimeric transcription factor activating protein-1 (AP-1) with c-Fos and mediates lung inflammatory response [44,60]. GSK3β phosphorylates MEKK1 through physical binding, and the activated MEKK1 further phosphorylates JNKK1 to activate JNK [52]. NF-κB is a master regulator in the host inflammatory response to harmful stimuli, which remains inactive in the cytoplasm by binding to the IκB. After the phosphorylation and degradation of IκB, the NF-κB dimer moves to the nucleus and activates the transcription of proinflammatory cytokines, thus promoting pulmonary inflammation [35,61]. GSK3β has been found to directly phosphorylate NF-κB p65 or indirectly activate NF-κB by affecting the activity of IκB kinase [29,53,54]. The data of molecular docking demonstrated that KP had a high binding activity with GSK3β, JNK1, and NF-κB p65. Therefore, we hypothesized that KP might alleviate the *P. aeruginosa*-induced acute pneumonia by regulating the GSK3β/JNK/c-Jun and NF-κB signaling pathways. A mouse model of acute *P. aeruginosa* pneumonia and an in vitro macrophage infection model were carried out for further validation.

*Pseudomonas aeruginosa* acute infections induce the overproduction of multiple proinflammatory cytokines in the host, leading to severe tissue lesions [62]. The suppression of cytokine overproduction has become a major strategy for the treatment of pneumonia [45]. KP was previously reported to inhibit the production of several proinflammatory cytokines, including IL-1β, TNF, IL-6, IL-17, IL-18, and MIP-2 [19,63,64]. Among them, TNF is a key proinflammatory cytokine mediating inflammation and could enhance the lung inflammatory response induced by *P. aeruginosa* via the JNK and NF-κB signaling pathways [44]. IL-1β is promptly produced in response to infections, and decreased production of IL-1β was found to alleviate the *P. aeruginosa*-induced lung tissue damage and reduce the mortality of mice [65]. IL-6 is essential in triggering acute inflammation, which promotes the production of a variety of inflammatory mediators [66]. MIP-2 is a potent chemokine for neutrophil recruitment into the inflamed lung [67]. Our study demonstrated that KP could effectively inhibit the *P. aeruginosa*-induced production of TNF, IL-1β, IL-6, and MIP-2 in the lungs of mice and macrophages, thus attenuating the severity of lung tissue damage and reducing the risk of systemic inflammation.

Multiple PAMPs of *P. aeruginosa* can activate TLRs and trigger host innate immune responses [62]. GSK3β is a highly conserved and ubiquitously expressed serine/threonine kinase that is able to regulate the inflammatory response by promoting TLR signaling [49]. The GSK3β inhibitor SB216763 has been found to attenuate the corneal inflammation caused by *P. aeruginosa* and reduce the levels of IL-1β and IL-6 [31]. Moreover, KP was previously reported to effectively inhibit GSK3β activation in the treatment of many diseases, such as myocardial ischemia/reperfusion injury and heart failure [59,68]. Additionally, GSK3β can directly phosphorylate many inflammatory mediators, such as NF-κB, IKK, IκB, MEKK1, JNK, TBK1, and NRF2 [32,48,49,52,53,54]. The inhibition of GSK3β and its downstream pathways has become an important strategy to mitigate lung injury [50,51]. A previous study showed that the deletion of GSK3β inhibited the degradation of IκB kinase, which led to the reduced activation and nuclear translocation of NF-κB [30]. In addition, GSK3β inhibition has been reported to reduce the activation of JNK, c-Jun, and NF-κB [30,32]. Furthermore, JNK and NF-κB are essential to regulate the expression of the cytokines, including TNF, IL-1β, IL-6, and MIP-2, and their activation levels were positively correlated to the severity of the *P. aeruginosa*-induced lung damage [34,44,47]. Previous studies have shown that KP treatment attenuated neuroinflammation by decreasing the phosphorylation of JNK and the nuclear translocation of NF-κB in microglia [19]. KP was able to significantly inhibit the phosphorylation of JNK and p38, which in turn inhibited the expression of NFATc1 and attenuated the wear particle-induced inflammatory osteolysis [21]. Furthermore, KP was found to inhibit intestinal inflammatory responses by reducing the activation of the TLR4/NF-κB pathway [17]. Further studies revealed that KP dose-dependently increased the levels of the phosphorylation-mediated inactivation of GSK3β in the lungs of mice and macrophages during *P. aeruginosa* infection, leading to the significant suppression of JNK, c-Jun, and NF-κB p65 phosphorylation. Interestingly, in the in vivo experiments, GSK3β in the KPH and Dex groups exhibited a higher level of phosphorylation inactivation than that in uninfected (NT) mice. GSK3 is one of the most substrate-rich protein kinases in cells and was originally found to regulate glucose metabolism and energy homeostasis [69]. GSK3β is a well-studied isoform of GSK3, which has been found to be involved in a wide range of diseases, including metabolic diseases, inflammatory diseases, neurological disorders, cancer, and atherosclerosis [29,48,69,70,71,72]. Under normal physiological conditions, GSK3β activity is finely regulated by a variety of signaling pathways, such as the PI3K/AKT and Wnt/β-catenin pathways [29,48,73]. These pathways dynamically regulate the phosphorylation status of GSK3β according to intra- and extracellular signaling changes, maintaining a relative balance between activation and inactivation. We speculated that this balance might be disrupted by the bacterial infection in this study, and the drug treatment decreased GSK3β activation, which led to higher levels of GSK3β phosphorylation. These findings suggested that KP ameliorated the *P. aeruginosa*-induced acute pneumonia through the inhibition of the GSK3β/JNK/c-Jun signaling pathway and NF-κB activation. 

This study suggested an alternative strategy for the treatment of acute *P. aeruginosa* lung infection and might also provide a basis for the treatment and management under chronic inflammatory conditions, such as cystic fibrosis, bronchiectasis, and chronic obstructive pulmonary disease. Additionally, although KP has demonstrated inhibitory effects against a wide range of pathogens [27], we did not find significant bacteriostatic effects of KP against *P. aeruginosa* in vivo. The therapeutic effects exerted by KP against *P. aeruginosa* pneumonia were mainly attributed to the suppression of the host inflammatory response. This study also suggested a combination of KP and antibiotics in treating bacterial infections, in which KP might enhance the efficacy of antibiotics by modulating host immune response and reducing the dosage of antibiotics and the risk of the development of drug resistance. Future studies could explore the therapeutic efficacy of KP in combination with antibiotics in bacterial pneumonia.

## 4. Materials and Methods

### 4.1. Target Gene Analysis of KP

The KP targets were from the SwissTargetPrediction database (http://swisstargetprediction.ch/ (accessed on 11 February 2025)), the ETCM database (http://www.tcmip.cn/ETCM/ (accessed on 11 February 2025)), and the TCMSP database (https://www.tcmsp-e.com/ (accessed on 11 February 2025)) with the screening criteria of “Homo sapiens” and *p*-value > 0.1. The names of the target genes were gained from the UniProt database (https://www.uniprot.org/ (accessed on 11 February 2025)). Subsequently, the obtained target names of KP were integrated.

### 4.2. Target Gene Analysis of P. aeruginosa Pneumonia

The PharmGKB database (https://www.pharmgkb.org/ (accessed on 11 February 2025)), GeneCards database (https://www.genecards.org/ (accessed on 11 February 2025)), and OMIM database (https://www.omim.org/ (accessed on 11 February 2025)) were used to search the target genes of *P. aeruginosa* pneumonia with the keyword “*P. aeruginosa* pulmonary infection”. The obtained target genes were integrated.

The targets of KP and *P. aeruginosa* pneumonia were imported into Venny 2.1.0 (https://bioinfogp.cnb.csic.es/tools/venny/ (accessed on 11 February 2025)) to obtain the intersecting targets, which were regarded as the potential targets of KP for treating *P. aeruginosa* pneumonia.

### 4.3. PPI Network

The intersecting targets were input into the STRING database (https://cn.string-db.org/ (accessed on 11 February 2025)), “Organisms” was limited to “Homo sapiens”, “medium confidence (0.400)” was set, and the targets without connection to others were hidden. Finally, the PPI network was exported and visualized via Cytoscape 3.9.1 (Cytoscape Consortium, San Diego, CA, USA).

### 4.4. GO and KEGG Pathway Enrichment Analysis

To understand the functions of the intersecting targets and potential pathways regulated by KP, the intersecting targets were input into the DAVID database (https://david.ncifcrf.gov/ (accessed on 11 February 2025)) with the restriction of “Homo sapiens”. Subsequently, the top 20 items of the KEGG pathway and GO involving BP, CC, and MF were imported into the bioinformatics website (https://www.bioinformatics.com.cn/ (accessed on 11 February 2025)) in descending order of *p*-value for visualization.

### 4.5. Molecular Docking

The three-dimensional (3D) structure of KP was optimized for energy-minimized structure by the MM2 functionality of Chem3D 19.0.0.22 (PerkinElmer Informatics, Inc., Wellesley, MA, USA). The 3D structures of the target proteins, GSK3β, JNK1, c-Jun, and NF-κB p65, were acquired from the RCSB Protein Data Bank database (https://www.rcsb.org/ (accessed on 11 February 2025)), and water molecules and ligands were removed using PyMOL 2.4.0 (Schrödinger, Inc., New York, NY, USA). Subsequently, the protein molecules were hydrogen-modified and docked with the KP molecule by AutoDockTools 1.5.6 (Scripps Research Institute, San Diego, CA, USA). Finally, the docking results with the lowest binding energy were visualized by PyMOL 2.4.0.

### 4.6. Reagents

KP for in vivo and in vitro experiments was supplied by Macklin Biochemical Co., Ltd. (Cat#: K812225; Shanghai, China). The standard of KP was purchased from Shanghai Yuanye Biotechnology (Cat#: B21126; Shanghai, China). Dex was purchased from MedChemExpress (Cat#: HY-14648, Shanghai, China). Enhanced Cell Counting Kit 8 (CCK8) was purchased from Elabscience Biotechnology (Cat#: E-CK-A362; Wuhan, China). The TNF (EK0527), IL-6 (EK0411), IL-1β (EK0394), and MIP-2 (EK0452) ELISA Kits were purchased from Boster Biological Technology (Wuhan, China). The SteadyPure Universal RNA Extraction Kit (AG21017), Evo M-MLV Reverse Transcription Premixed Kit (AG11728), and SYBR Green Pro Taq HS Premixed qPCR Kit (AG11701) were purchased from Accurate Biotechnology (Hunan, China). Antibodies for β-actin (AF0003), BCA Protein Assay Kit (P0011), ECL substrate kit (P0018S), goat anti-rabbit (A0208), and anti-mouse (A0216) IgG were obtained from Beyotime Biotechnology (Shanghai, China). The antibodies for phospho-NF-κB p65 (3033), NF-κB p65 (8242), phospho-GSK3β (Ser9) (5558), GSK3β (12456), phospho- JNK (9251), JNK (9252), phospho-c-Jun (Ser73) (3270), and c-Jun (9165) were supplied by Cell Signaling Technology (Danvers, MA, USA). 

### 4.7. Determination of KP Content

The composition and content of the KP sample were determined by high-performance liquid chromatogram (HPLC). The sample and standard of KP were dissolved in acetonitrile and filtered through a microporous membrane, respectively. Chromatographic conditions: the chromatographic column was SHIMSEN Superb IIC18 (4.6 × 250 mm, 5 μm); the mobile phase was acetonitrile:water = 32:68; the column temperature was 30 °C; the flow rate was 1.00 mL/min; the injection volume was 10 μL; and the detection wavelength of the UV detector (Agilent 1260 liquid chromatography system, Agilent Technologies, Inc., Santa Clara, CA, USA) was 265 nm.

**Figure 9:**
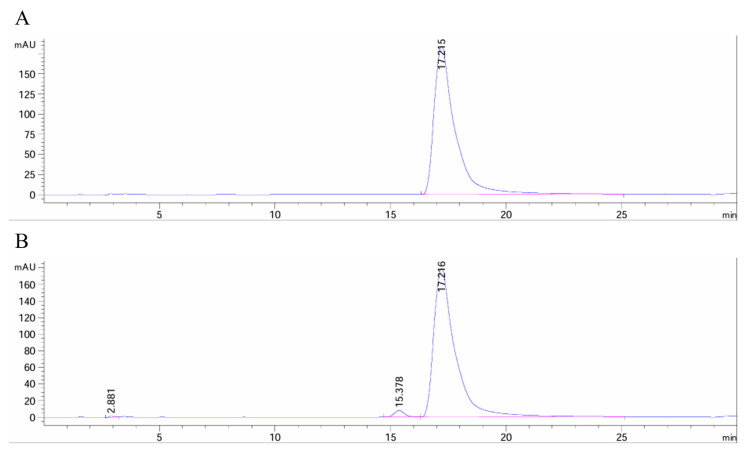
High-performance liquid chromatogram of the KP standard (**A**) and sample (**B**). The pink line indicated the baseline, and the blue line represented the detected signal above the baseline. Peaks were labeled with their respective retention times.

According to the HPLC graph, the KP sample and standard had the same retention time under the same chromatographic conditions (Figure 9). In addition, the integration result displayed that the relative peak area of the KP sample was 97.85%, which indicated a high purity of the KP sample.

### 4.8. Bacterial Culture

The *P. aeruginosa* strain PAO1 was utilized in the experiments. Briefly, bacteria were cultured in LB broth with shaking at 37 °C in the early stationary phase. After washing with PBS and centrifuging at 13,000 rpm for 3 min, the bacteria were resuspended for subsequent experiments.

### 4.9. Animals and Drug Treatment

The male C57BL/6N mice (Charles River Laboratories, Beijing, China) aged 8 weeks with a body weight of 22 ± 2 g were acclimatized in a specific pathogen-free (SPF) facility for one week before the experimental research was conducted. The animal protocol has been approved by the Animal Ethics Committee of Shandong University of Traditional Chinese Medicine (protocol number: SDUTCM20240716002).

The mice were randomly divided into 5 groups, each of which contained 8 mice, including the NT, Saline, KPL (10 mg/kg), KPH (30 mg/kg), and Dex (2.5 mg/kg) groups. All mice were administered with drugs or saline via intraperitoneal injection. The mice in the NT group received an intranasal simulation with saline, whereas the mice from the other groups were intranasally administered with PAO1. Drugs were given 1 h before the infection, and a subsequent dose was administered 8 h after the first dose.

### 4.10. Lung Infection and Tissue Processing

The mice from the Saline, KPL, KPH, and Dex groups received 5 × 10^8^ colony-forming unit (CFU) of PAO1 per mouse through intranasal infection, while the mice in the NT group were given an equal volume of saline. Subsequently, the mice were euthanized after 24 h of infection, and BALF was obtained by lung lavage with 1 mL PBS. For CFU counting, 10 μL BALF was spread onto an LB agar plate, followed by 24 h incubation at 37 °C. The supernatants for cytokine detection were collected from the remaining BALF after centrifugation at 1200 rpm for 5 min at 4 °C. After erythrocytes were lysed, the BALF cells were lysed with 250 μL of 0.5% cetyltrimethylammonium chloride (CTAC) and then centrifuged to collect the supernatants for MPO assay.

The left lungs were ground in HEPES buffer (50 mM; 4 μL per 1 mg lung) to obtain lung homogenates. For CFU counting, 10 μL homogenates were applied onto an LB agar plate, followed by 24 h incubation at 37 °C. Subsequently, the remaining homogenates were centrifuged at 13,000 rpm for 10 min at 4 °C, and the supernatants were collected for cytokine detection. The pellets were homogenized in 0.5% CTAC (4 μL per 1 mg lung) and centrifuged to obtain extracts for measuring MPO activity.

### 4.11. Survival Study and Disease Scores

A survival study was applied to explore the therapeutic efficacy of KP and disease progression, which was based on a 21-point scoring system [35]. Briefly, the mice were administered 1 × 10^9^ CFU/mouse of PAO1 intranasally. The drugs or saline were administered on the infection day and day 1 post-infection. Subsequently, the survival rates and disease scores of mice were recorded daily for 10 days after infection. The detailed scoring system was shown in Table 4.

### 4.12. Histological Assessment

The post-caval lobes of mouse lung tissues were soaked in 10% formalin for 2 days. Subsequently, the lung tissues were dehydrated with ascending gradients of ethanol, transparently treated with xylene, and embedded in paraffin. The tissues were then sectioned, treated with xylene to remove the paraffin, and rehydrated with descending gradients of ethanol. Finally, the tissue sections were subjected to H&E staining. The images of the lung sections were observed and photographed at 100× or 400× magnification using a microscope (Olympus IX73, Olympus Corporation, Tokyo, Japan).

The score of lung injury was evaluated histologically according to a previously reported scoring criterion [74]. Briefly, at 400× magnification, 20 random non-repeating fields of view were sought and scored according to neutrophils in the alveolar space, neutrophils in the interstitial space, hyaline membranes, proteinaceous debris filling the airspaces, and alveolar septal thickening. Specifically, scoring was based on the following guidelines: (A) the number of neutrophils in the alveolar space (0:none, 1:1–5, 2:>5); (B) the number of neutrophils in the interstitial space (0:none, 1:1–5, 2:>5); (C) the number of hyaline membranes (0:none, 1:1, 2:>1); ( D) the extent to the filling of airspace with protein fragments (0: none, 1:1, 2:>1); (E) multiples of alveolar septal thickening (0:<2×, 1:2×–4×, 2:>4×). Scoring formula: score = [(20 × A) + (14 × B) + (7 × C) + (7 × D) + (2 × E)]/(number of fields × 100). Finally, the histologic score for each sample was calculated by the provided formula.

### 4.13. Protein Concentration Assay

The total protein concentration of the BALF and lung supernatants of mice was detected by BCA assay. Briefly, 10 μL samples were mixed with 200 μL BCA reagents and incubated at 37 °C for 30 min. Subsequently, the OD was measured at 562 nm, and the total protein concentration of each sample was calculated based on the standard curve and dilution factors.

### 4.14. MPO Assay

The neutrophil infiltration in the lungs of mice was evaluated by MPO assay [34]. In brief, 75 μL lysates from BALF cells or lung tissues were mixed with 75 μL substrate, a mixture of 2.2 mM H_2_O_2_, 3 mM TMB dihydrochloride, and 120 μM resorcinol, and the same volume of deionized water mixed with the substrate was used as a blank control. After incubation at room temperature for 2 min, the reaction was terminated with 150 μL H_2_SO_4_ (2 M), and the OD was measured at 450 nm.

### 4.15. Macrophage Culture and Infection

The bone marrow cells from the mouse femur and tibia were cultured with DMEM medium containing 1% penicillin–streptomycin, 10 ng/mL macrophage colony-stimulating factor, and 10% FBS. The culture medium was half-replaced every 2–3 days. On day 7, the DMEM medium with only 10% FBS was used for the following experiments. After the pretreatment with KP for 1 h, the cells were infected with PAO1 (MOI: 10) or mock-infected (NT) with PBS. Furthermore, the cell pellets were lysed for Western blot analysis of p-GSK3β at Ser9, GSK3β, p-JNK, JNK, p-c-Jun, c-Jun, p-NF-κB p65, and NF-κB p65 at 1 h after infection, whereas the cell-free supernatants were obtained for cytokine detection at 3 h, 6h, and 12h after infection.

### 4.16. Cell Viability Assay

Cells were seeded at a density of 1 × 10^4^ per well in a 96-well plate and treated with KP at concentrations of 0, 25, 50, 100, or 120 μM. After incubation at 37 °C for 12 h, 10 μL CCK-8 solution was added to each well, and the incubation was continued for an additional 4 h. The OD was measured at 450 nm.

### 4.17. Cytokine Analysis

The concentrations of TNF, IL-6, IL-1β, and MIP-2 in the lung tissues, BALF or cell-free supernatants were examined by ELISA, and the experimental processes were conducted according to the manufacturer’s instructions.

### 4.18. Real-Time Quantitative PCR (RT-qPCR)

The mRNA transcription levels of *GSK3β*, *JNK1*, *c-Jun*, and *p65 (RelA)* were examined by RT-qPCR. The total RNA extraction, reverse transcription, and qPCR analyses of lung tissues and cells were performed using SteadyPure Universal RNA Extraction Kit, Evo M-MLV Reverse Transcription Premixed Kit, and SYBR Green Pro Taq HS Premixed qPCR Kit, respectively. The experimental processes were conducted according to the manufacturer’s instructions. β-actin served as the endogenous control. Primer sequences were listed in Table 5.

### 4.19. Western Blotting

The phosphorylation levels of GSK3β, JNK, c-Jun, and NF-κB p65 were analyzed. The cells and right lungs of mice were lysed in radioimmunoprecipitation assay (RIPA) buffer for 30 min and then centrifuged to obtain the lysates at 4 °C. The cell or lung lysates were collected to denature at 95 °C for 5 min. The proteins were separated using electrophoresis in 10% SDS polyacrylamide gel and transferred to a 0.45 μm polyvinylidene difluoride (PVDF) membrane. Subsequently, the membrane was blocked with 5% skim milk at room temperature for 1.5 h and incubated overnight with primary antibody. The next day, the washed membrane was probed with a secondary antibody for 2 h. Finally, the ECL substrate was applied onto the membrane, and images were generated with the Vilber Lourmat Fusion FX imaging system (Vilber Lourmat, Marne La Vallée, France).

### 4.20. Statistical Analysis

Statistical analysis was conducted using GraphPad Prism 9.5.0. Data are expressed as mean ± standard deviation. One-way analysis of variance (ANOVA) with Dunnett’s multiple comparisons test was applied for multiple group comparisons. A *p*-value < 0.05 was regarded as statistically significant.

## 5. Conclusions

Altogether, this research first applied network pharmacology to explore the underlying mechanisms of KP in treating *P. aeruginosa* pneumonia and validated them through in vivo and in vitro studies. The results of network pharmacology suggested that KP might treat *P. aeruginosa* pneumonia by modulating GSK3β/JNK/c-Jun signaling pathway and NF-κB. Further experimental results proved that KP reduced the production of proinflammatory cytokines by inhibiting GSK3β/JNK/c-Jun signaling pathways and NF-κB activation, which effectively mitigated the *P. aeruginosa*-induced acute lung inflammation and injury and elevated the survival rates of mice. In the future, we will focus on evaluating the synergistic therapeutic potential of KP in combination with antibiotics.

## Figures and Tables

**Table 1:** The top 25 targets of KP on *P. aeruginosa* pulmonary infection in the PPI network.

Number	UniProt ID	Target Name	Degree
1	P01375	TNF	60
2	P00533	EGFR	52
3	P31749	AKT1	51
4	P10415	BCL2	50
5	P14780	MMP9	49
6	P05412	JUN	47
7	P42574	CASP3	47
8	P12931	SRC	45
9	P35354	PTGS2	45
10	P03372	ESR1	44
11	P07900	HSP90AA1	43
12	P37231	PPARG	42
13	P49841	GSK3B	38
14	P05362	ICAM1	36
15	P09601	HMOX1	36
16	P08238	HSP90AB1	36
17	P35968	KDR	35
18	Q04206	RELA	34
19	P42224	STAT1	34
20	P08253	MMP2	34
21	P19320	VCAM1	33
22	P45983	MAPK8	31
23	P09874	PARP1	30
24	P05067	APP	29
25	P08069	IGF1R	29

**Table 2:** The top 20 KEGG-enriched pathways sorted by *p*-value.

ID	Description	Gene Ratio	*p*-Value	Count
hsa05200	Pathways in cancer	40.54	4.91 × 10^−18^	30
hsa05417	Lipid and atherosclerosis	28.38	9.89 × 10^−17^	21
hsa05418	Fluid shear stress and atherosclerosis	27.03	4.22 × 10^−19^	20
hsa05022	Pathways of neurodegeneration—multiple diseases	21.62	3.28 × 10^−6^	16
hsa04933	AGE-RAGE signaling pathway in diabetic complications	20.27	2.37 × 10^−14^	15
hsa05167	Kaposi sarcoma-associated herpesvirus infection	20.27	2.33 × 10^−10^	15
hsa04151	PI3K-Akt signaling pathway	20.27	5.04 × 10^−7^	15
hsa05010	Alzheimer disease	20.27	1.34 × 10^−6^	15
hsa04657	IL-17 signaling pathway	18.92	2.58 × 10^−13^	14
hsa05161	Hepatitis B	18.92	2.96 × 10^−10^	14
hsa05207	Chemical carcinogenesis—receptor activation	18.92	8.13 × 10^−9^	14
hsa01522	Endocrine resistance	17.57	1.03 × 10^−11^	13
hsa04668	TNF signaling pathway	17.57	6.37 × 10^−11^	13
hsa05169	Epstein–Barr virus infection	17.57	4.67 × 10^−8^	13
hsa05208	Chemical carcinogenesis—reactive oxygen species	17.57	1.39 × 10^−7^	13
hsa05163	Human cytomegalovirus infection	17.57	1.53 × 10^−7^	13
hsa04010	MAPK signaling pathway	17.57	3.53 × 10^−6^	13
hsa04926	Relaxin signaling pathway	16.22	4.17 × 10^−9^	12
hsa04915	Estrogen signaling pathway	16.22	8.54 × 10^−9^	12
hsa05152	Tuberculosis	16.22	1.35 × 10^−7^	12

**Table 3:** The molecular docking of KP with target proteins.

Target Name	PDB ID	Protein	Binding Energy (kcal/mol)
GSK3B	1Q5K	GSK3β	−5.44
MAPK8	3PZE	JNK1	−5.03
JUN	5FV8	c-Jun	−4.18
RELA	1IKN	NF-κB p65	−5.56

**Table 4:** The scoring rules of the disease status of mice.

Score	0	1	2	3
Hair feature	Normal	Lack of grooming	Rough	Very obvious rough
Body posture	Normal	Hunched sitting position	A hunched stance with the head lying on the ground	Prone and unable to stay in an upright posture
Activity/Behavior	Normal	Minor changes in behavior	Decreased activity with increased respiratory rate	Only moves when stimulated
Appetite	Normal	Reduced appetite	Not eating food after the last checkpoint	Not eating food for the last two checkpoints
Hydration	Normal	Mild dehydration	Moderate dehydration	Severe dehydration
Change in weight compared with pre-infection	<5.0%	<10.0%	10.0–15.0%	>15.0%
Body temperature (ventral surface temperature)	33–34 °C	28–32.5 °C	25–27.5 °C	<24.5 °C

Total possible score = 21; Endpoint for euthanasia > 15.

**Table 5:** Forward and backward primer sequences for RT-qPCR analysis.

Name	Primer Sequence (5′ → 3′)
*GSK3β*	F: CCCTCCACATGCTCGGATTCA
	R: ATTGGTCTGTCCACGGTCTCC
*JNK1*	F: AGAAACTGTTCCCCGATGTGC
	R: GCTGGAGAGCTTCATCTACGGA
*c-Jun*	F: AAACTCCGAGCTGGCATCCAC
	R: GCTGCGTTAGCATGAGTTGGC
*p65 (RelA)*	F: TCGCCACCGGATTGAAGAGAA
	R: CGGGGTTCAGTTGGTCCATTG
*β* *-actin*	F: CATCCGTAAAGACCTCTATGCCAAC
	R: ATGGAGCCACCGATCCACA

## Data Availability

Data will be made available on request.

## Supplementary Materials

The following supporting information can be downloaded at: https://www.mdpi.com/article/10.3390/ph18030322/s1. The results of the effect of KP on proinflammatory cytokine production in the lungs of uninfected mice were shown in Figure S1. The results of the effect of KP on the gene expression of GSK3β, JNK, c-Jun, and NF-κB p65 in the lungs of mice and BMDMs infected with *P. aeruginosa* were shown in Figure S2 and Figure S3, respectively.
