# Supplementary material for: Kaempferol Mitigates Pseudomonas aeruginosa-Induced Acute Lung Inflammation Through Suppressing GSK3β/JNK/c-Jun Signaling Pathway and NF-κB Activation
Source: Pharmaceuticals (Basel). 2025 Feb 25;18(3):322. doi: 10.3390/ph18030322 (PMC11944347; doi:10.3390/ph18030322)
Supplement: Supplementary file 1 [file pharmaceuticals-18-00322-s001.zip › Original Western blots.pdf]

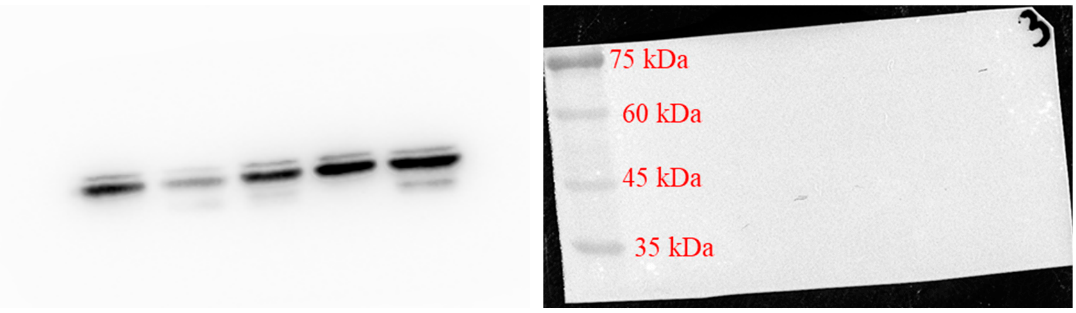

Western blotting analysis for p-GSK3β (Ser9) (lung tissue sample 1).

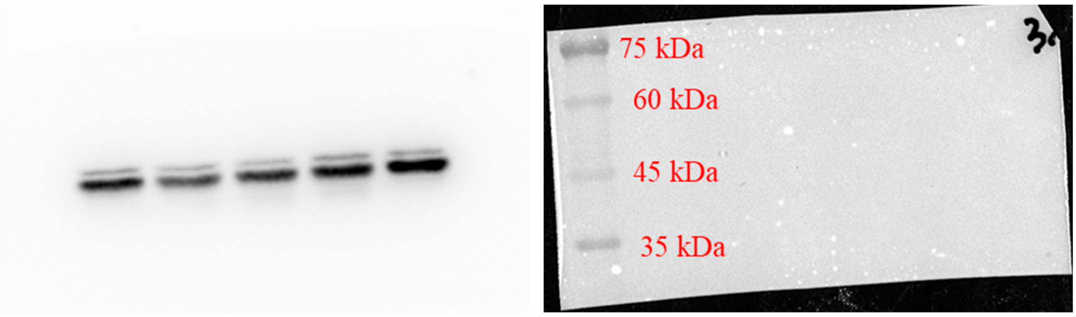

Western blotting analysis for p-GSK3β (Ser9) (lung tissue sample 2).

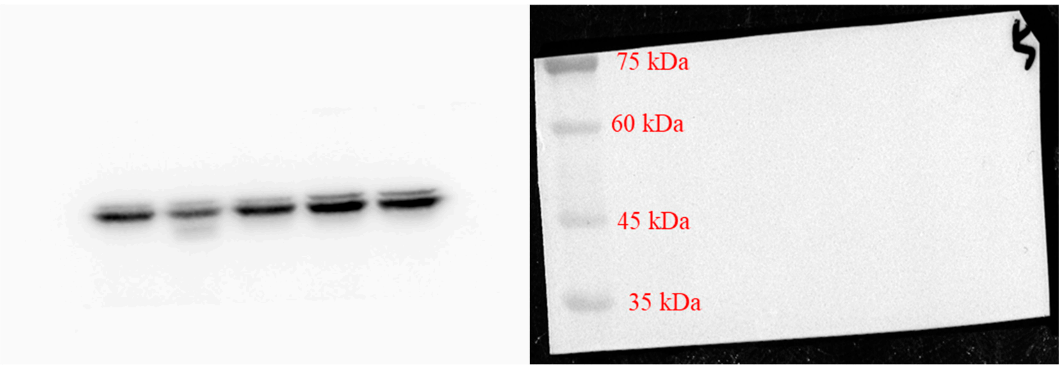

Western blotting analysis for p-GSK3β (Ser9) (lung tissue sample 3).

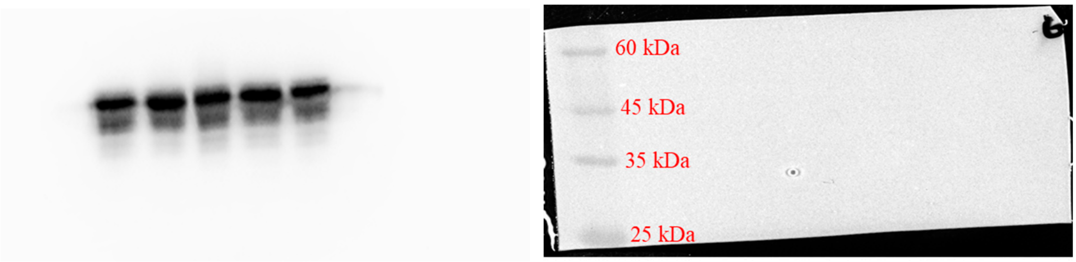

Western blotting analysis for GSK3β (lung tissue sample 1).

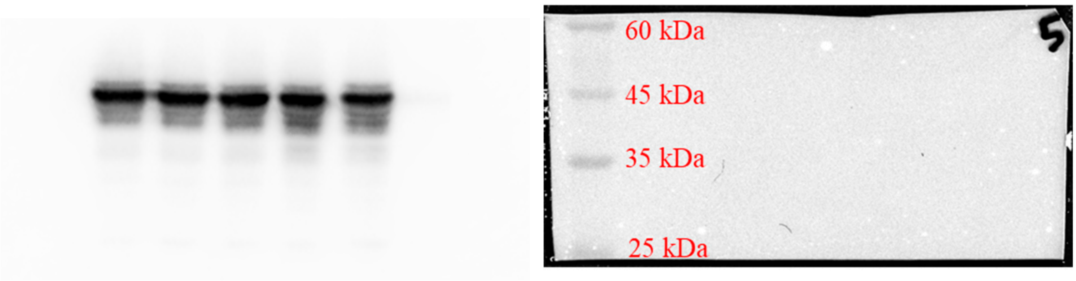

Western blotting analysis for GSK3β (lung tissue sample 2).

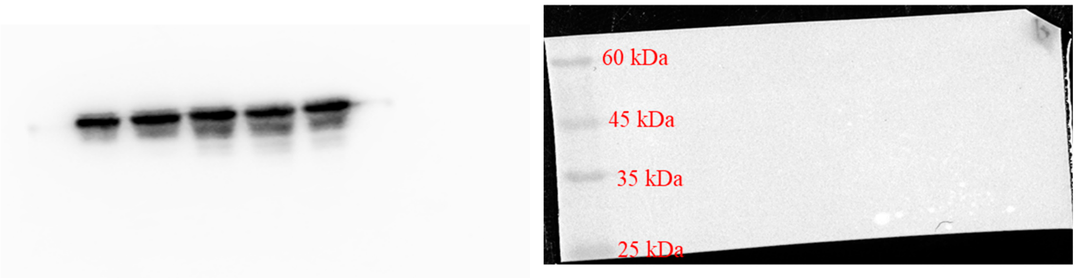

Western blotting analysis for GSK3β (lung tissue sample 3).

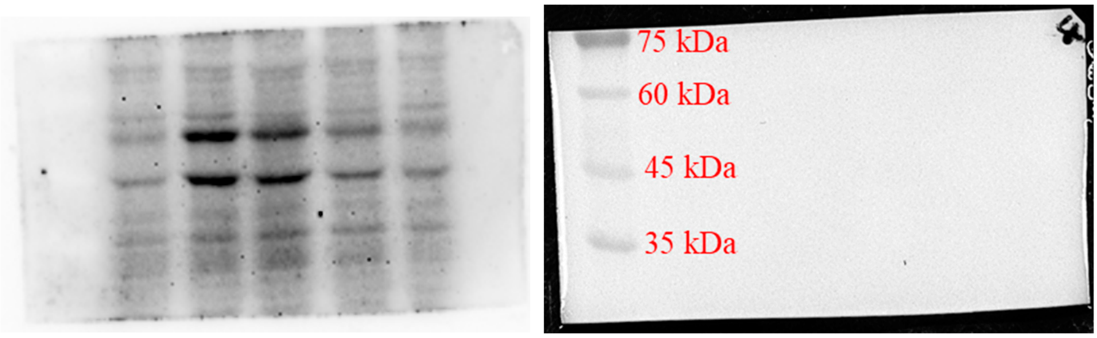

Western blotting analysis for p-JNK (lung tissue sample 1).

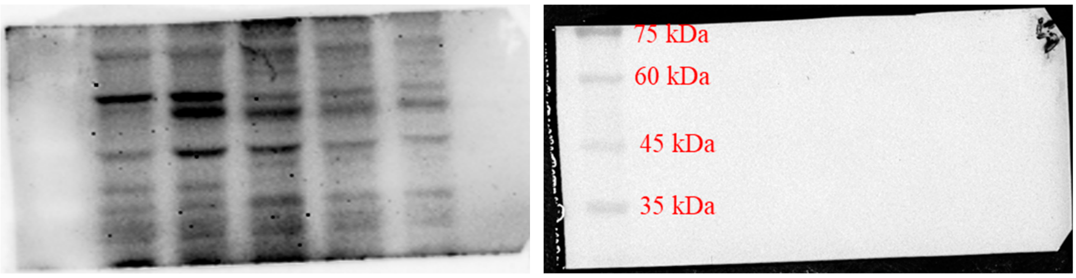

Western blotting analysis for p-JNK (lung tissue sample 2).

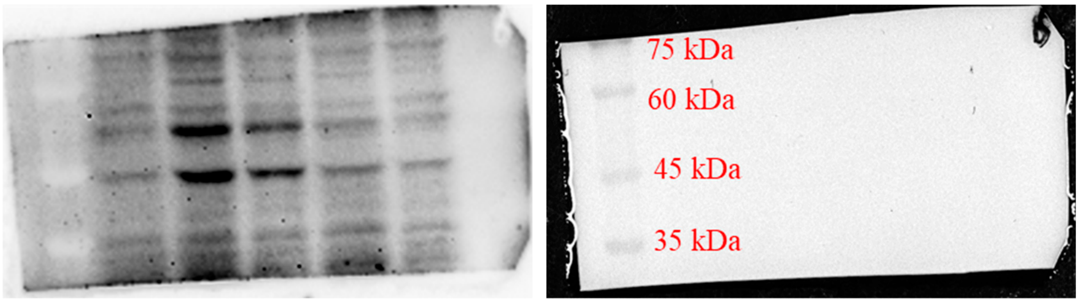

Western blotting analysis for p-JNK (lung tissue sample 3).

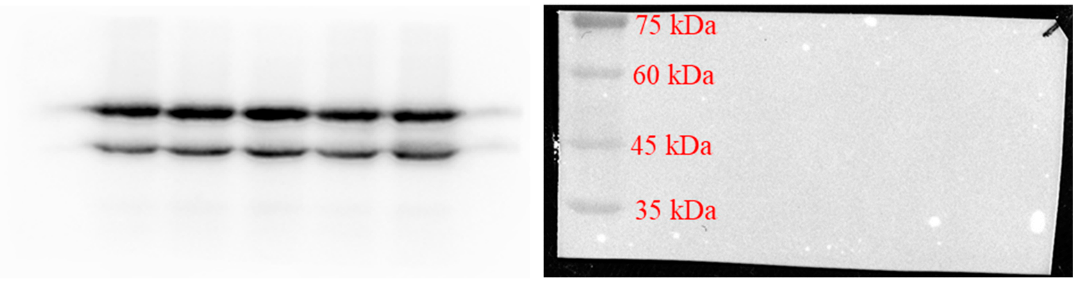

Western blotting analysis for JNK (lung tissue sample 1).

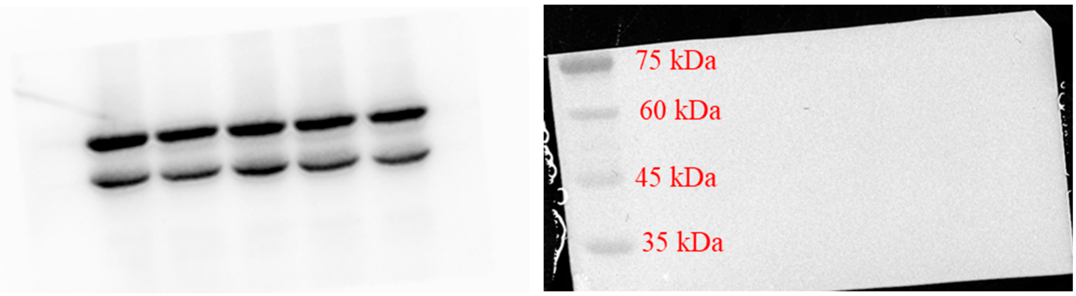

Western blotting analysis for p-JNK (lung tissue sample 2).

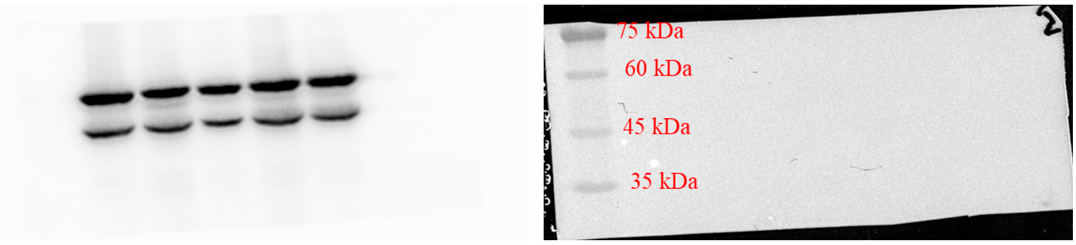

Western blotting analysis for p-JNK (lung tissue sample 3).

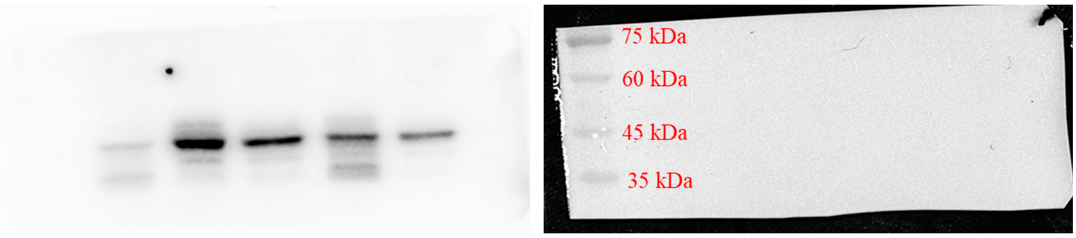

Western blotting analysis for p-c-Jun (lung tissue sample 1).

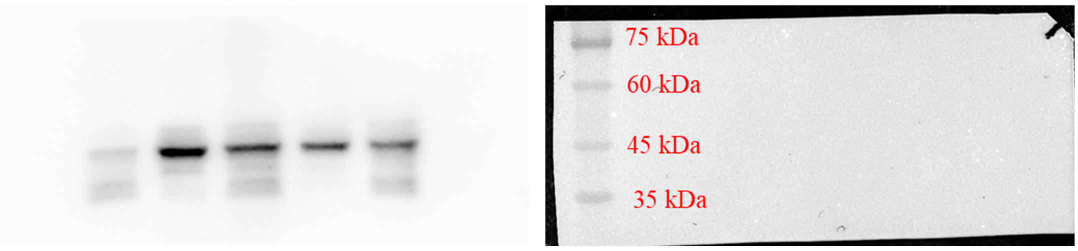

Western blotting analysis for p-c-Jun (lung tissue sample 2).

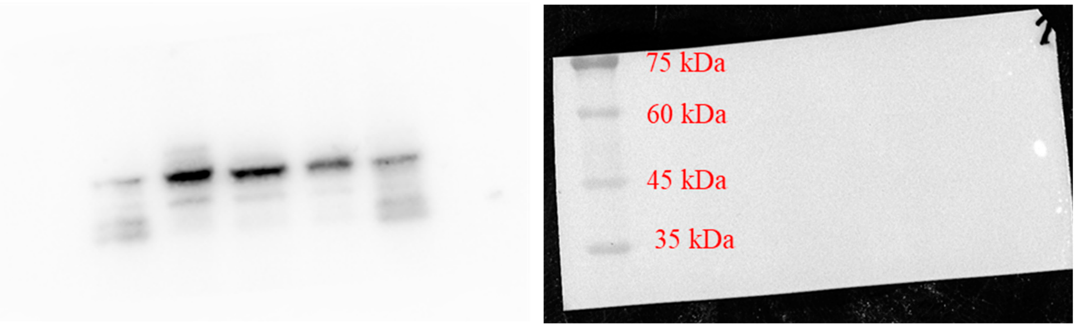

Western blotting analysis for p-c-Jun (lung tissue sample 3).

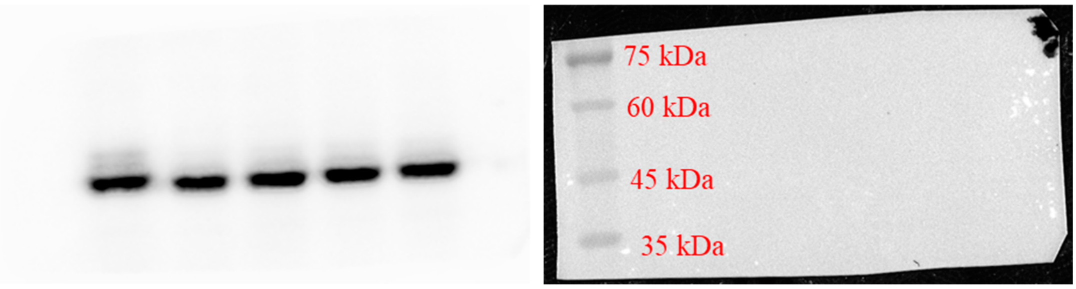

Western blotting analysis for c-Jun (lung tissue sample 1).

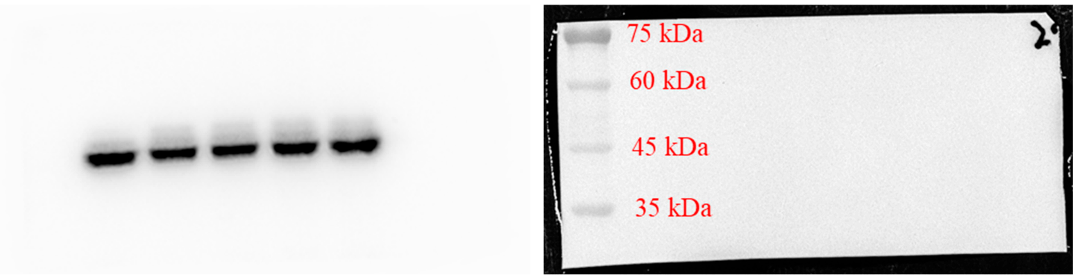

Western blotting analysis for c-Jun (lung tissue sample 2).

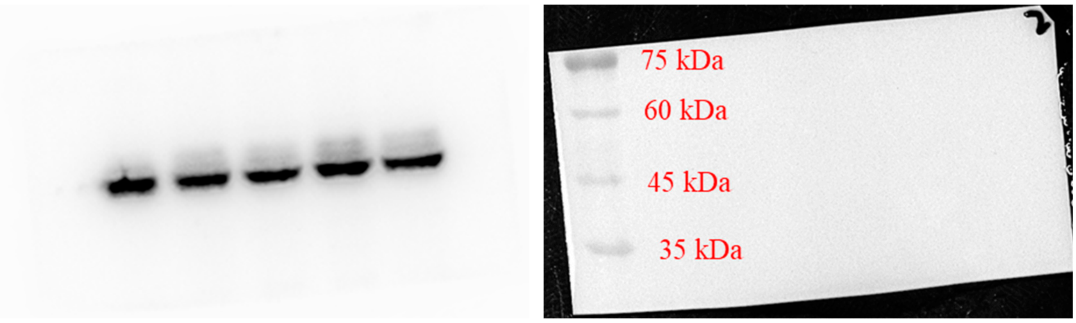

Western blotting analysis for c-Jun (lung tissue sample 3).

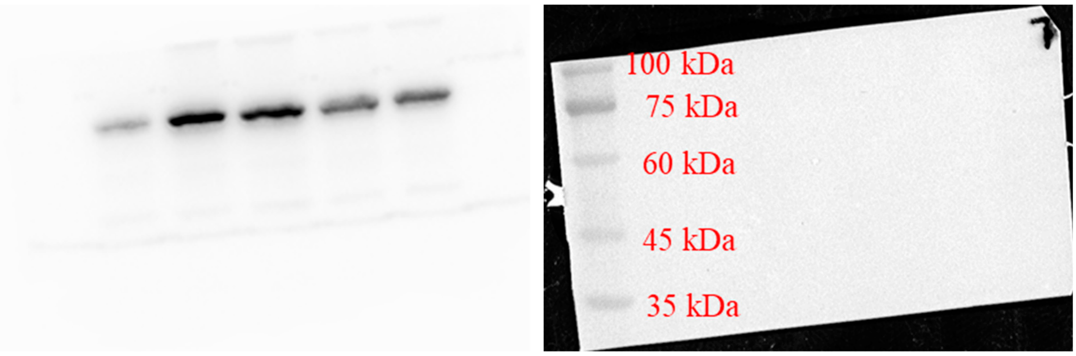

Western blotting analysis for p-p65 (lung tissue sample 1).

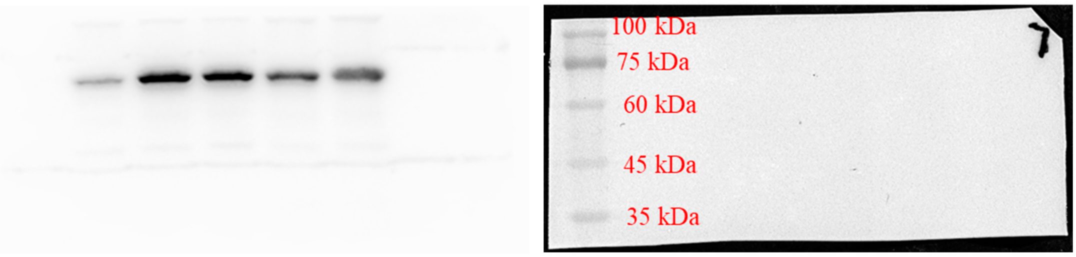

Western blotting analysis for p-p65 (lung tissue sample 2).

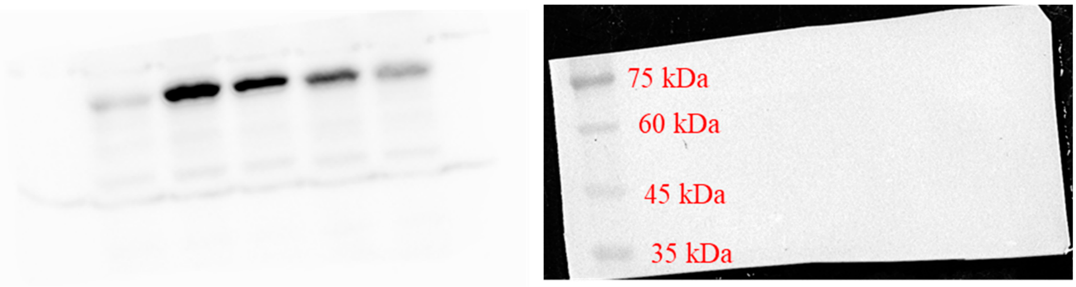

Western blotting analysis for p-p65 (lung tissue sample 3).

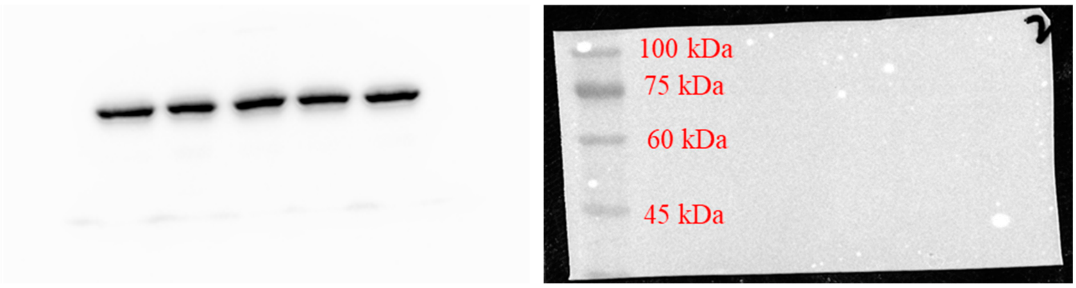

Western blotting analysis for p65 (lung tissue sample 1).

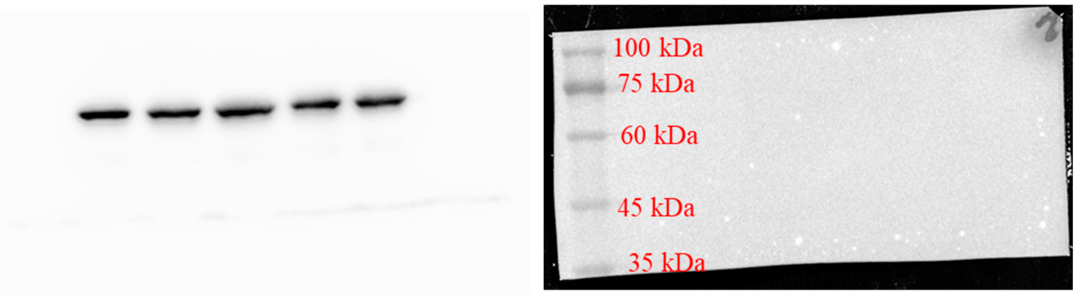

Western blotting analysis for p65 (lung tissue sample 2).

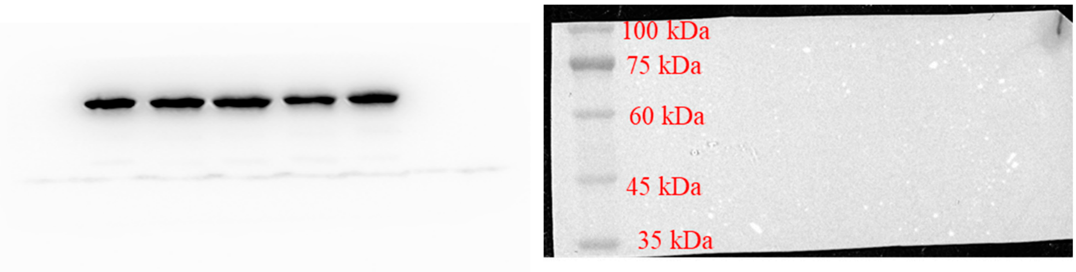

Western blotting analysis for p65 (lung tissue sample 3).

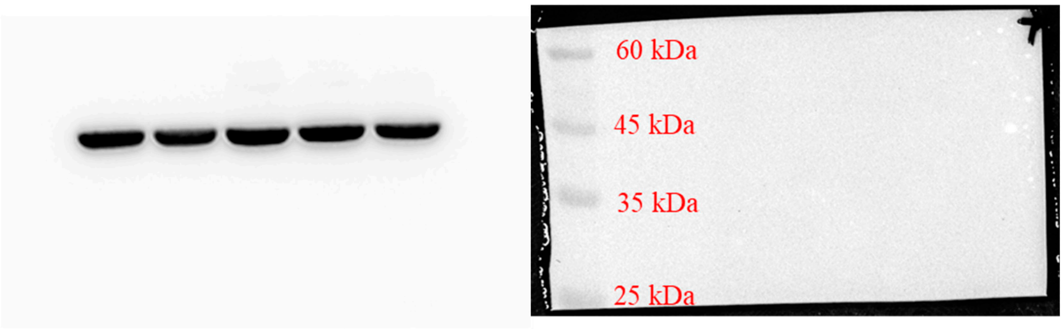

Western blotting analysis for β-actin (lung tissue sample 1).

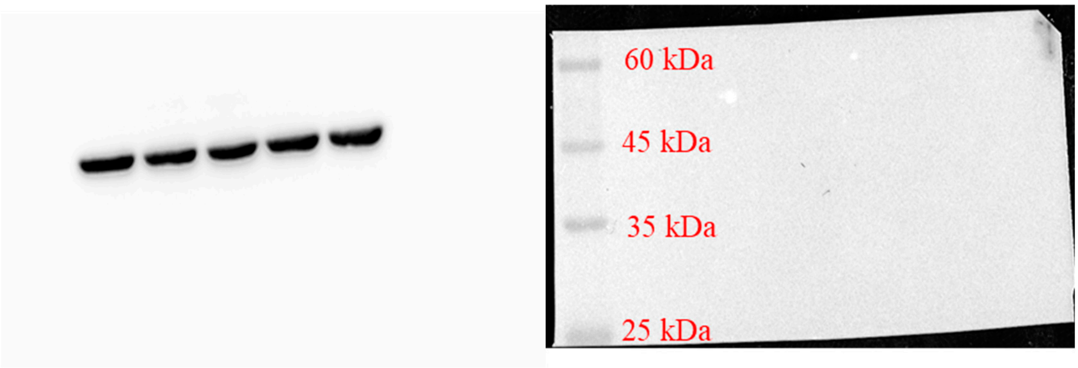

Western blotting analysis for β-actin (lung tissue sample 2).

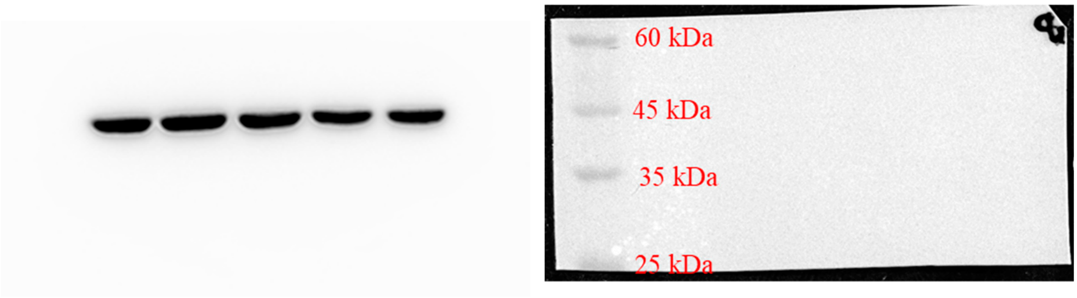

Western blotting analysis for β-actin (lung tissue sample 3).

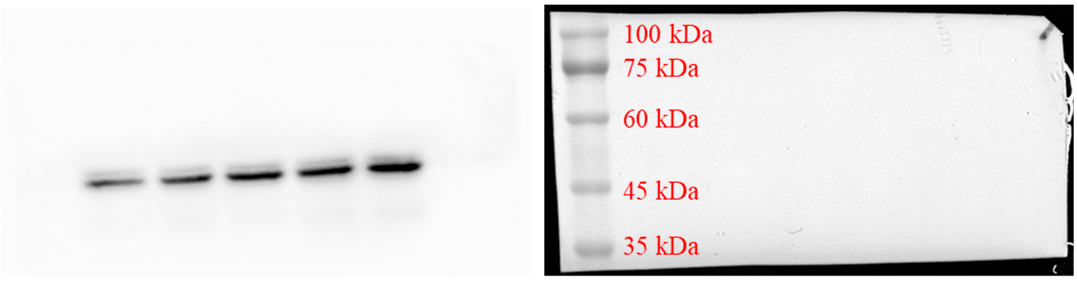

Western blotting analysis for p-GSK3β (Ser9) (cell sample 1).

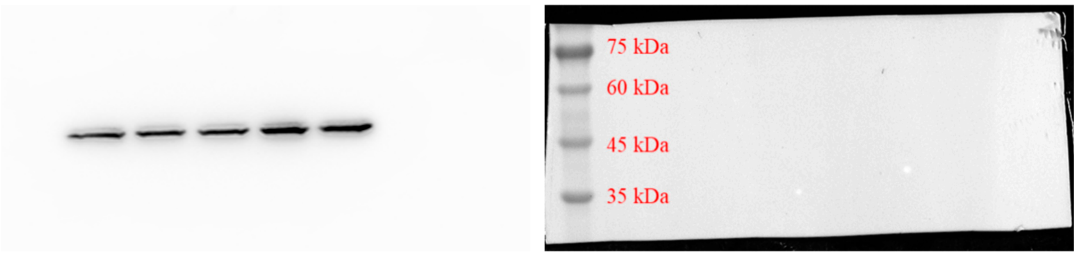

Western blotting analysis for p-GSK3β (Ser9) (cell sample 2).

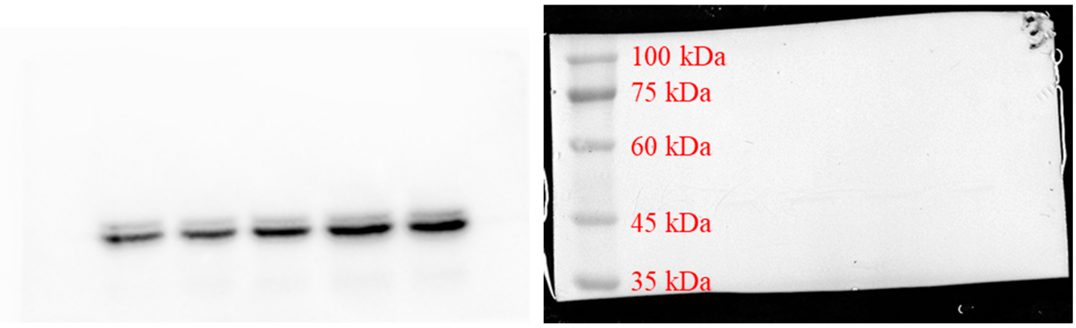

Western blotting analysis for p-GSK3β (Ser9) (cell sample 3).

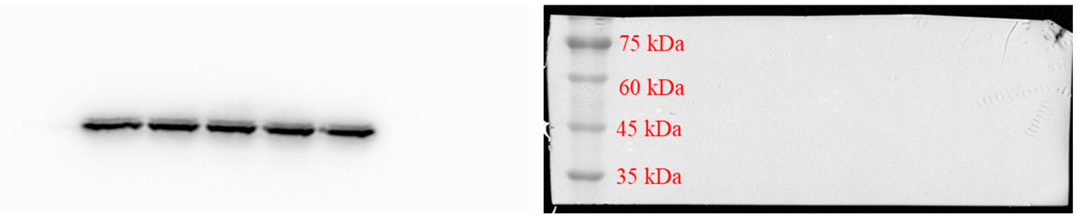

Western blotting analysis for GSK3β (cell sample 1).

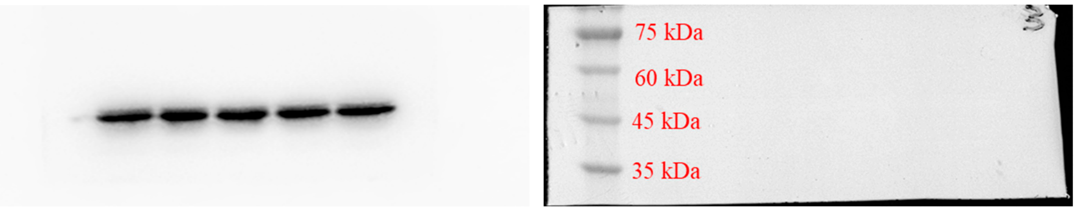

Western blotting analysis for GSK3β (cell sample 2).

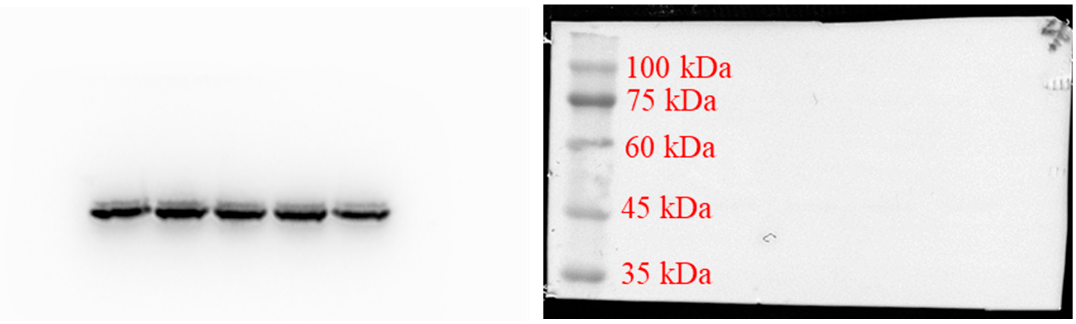

Western blotting analysis for GSK3β (cell sample 3).

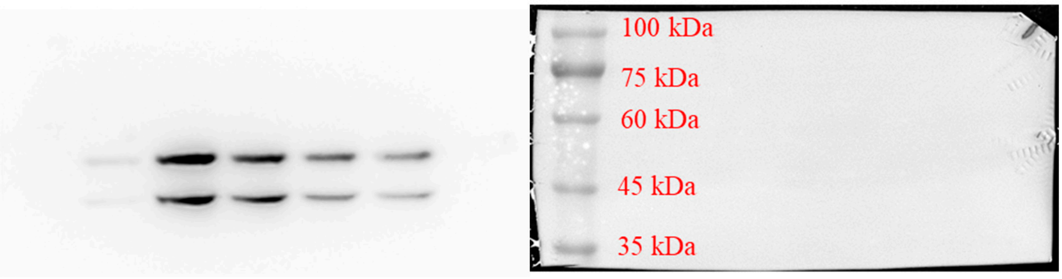

Western blotting analysis for p-JNK (cell sample 1).

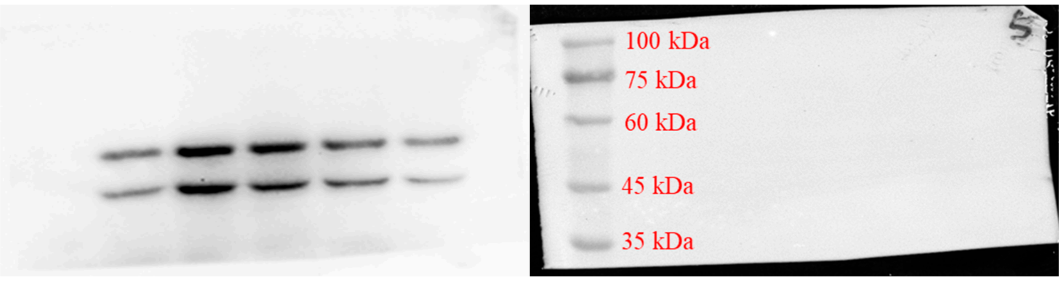

Western blotting analysis for p-JNK (cell sample 2).

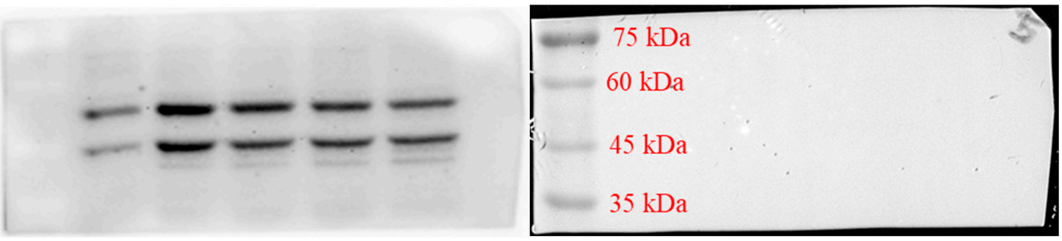

Western blotting analysis for p-JNK (cell sample 3).

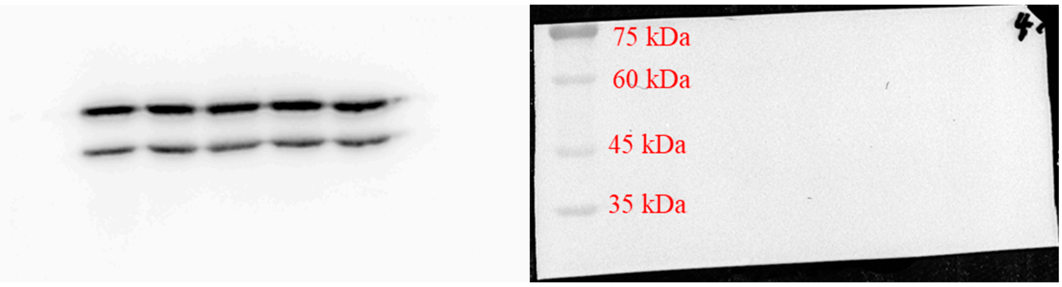

Western blotting analysis for JNK (cell sample 1).

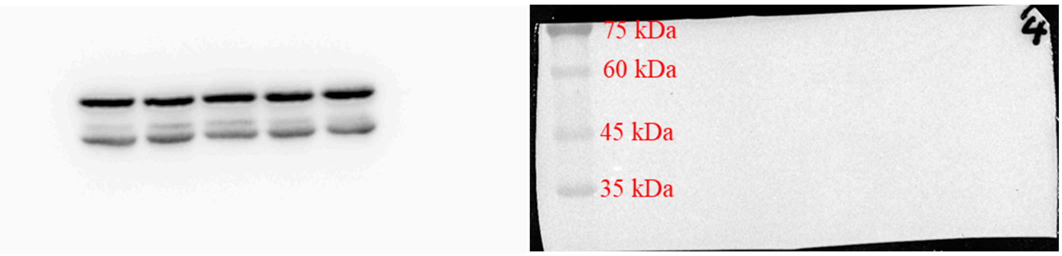

Western blotting analysis for JNK (cell sample 2).

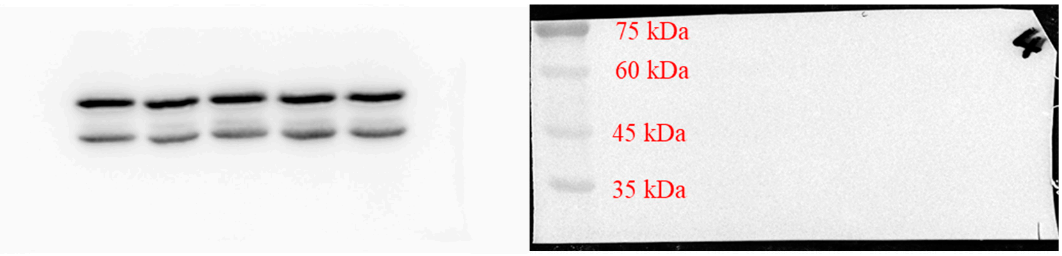

Western blotting analysis for JNK (cell sample 3).

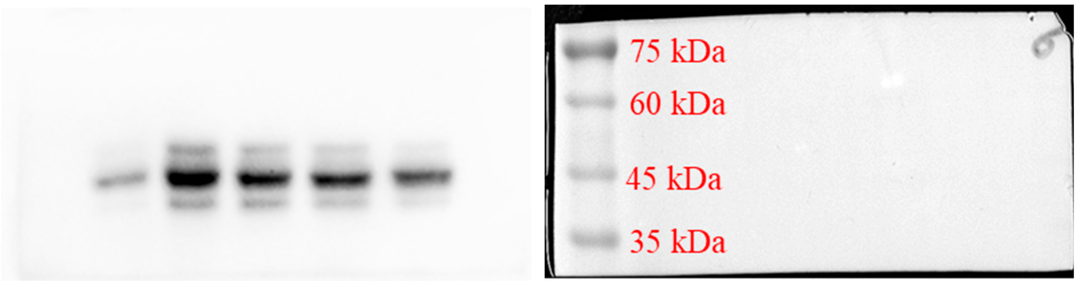

Western blotting analysis for p-c-Jun (cell sample 1).

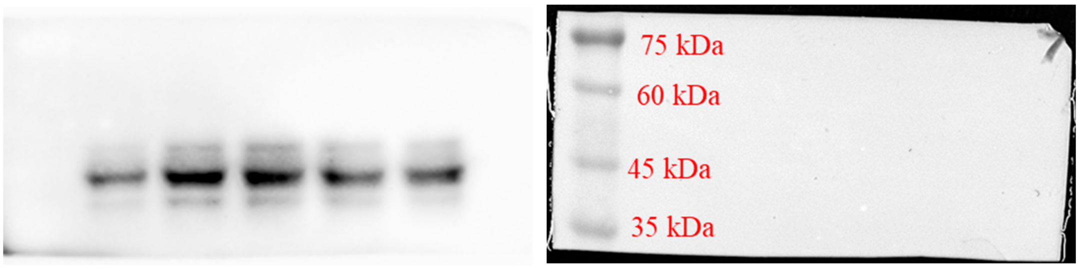

Western blotting analysis for p-c-Jun (cell sample 2).

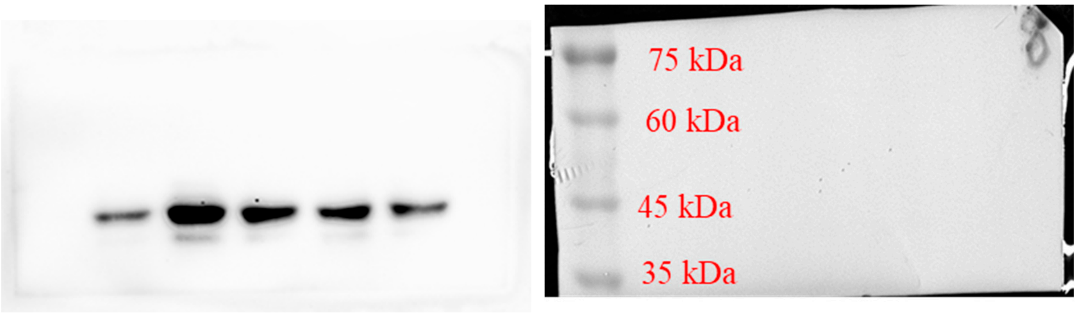

Western blotting analysis for p-c-Jun (cell sample 3).

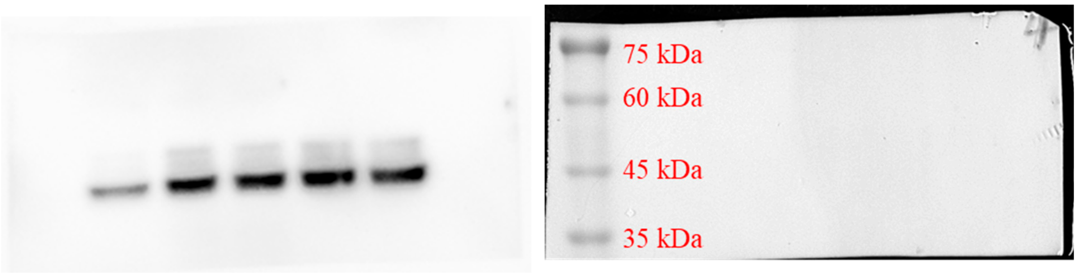

Western blotting analysis for c-Jun (cell sample 1).

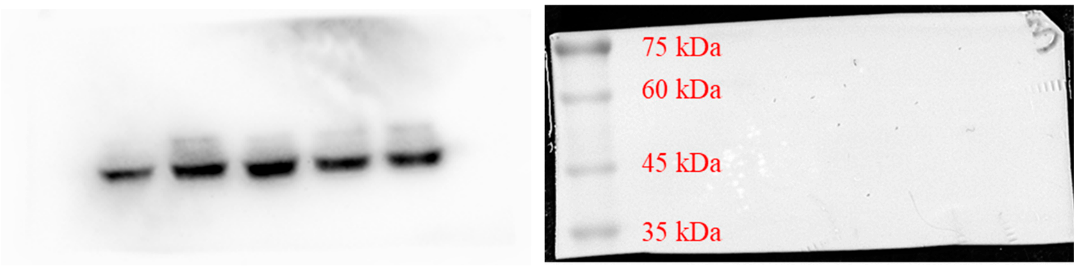

Western blotting analysis for c-Jun (cell sample 2).

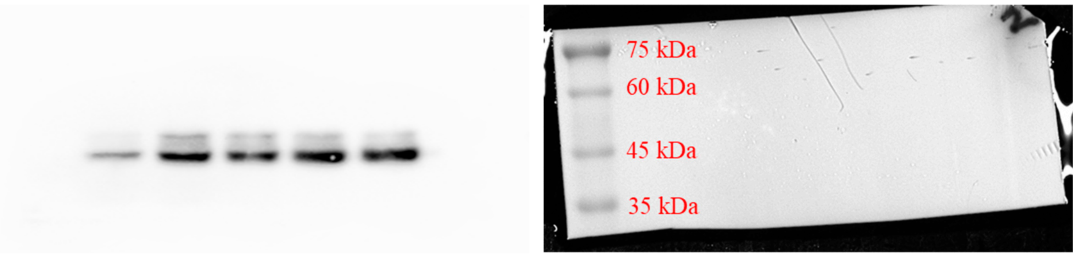

Western blotting analysis for c-Jun (cell sample 3).

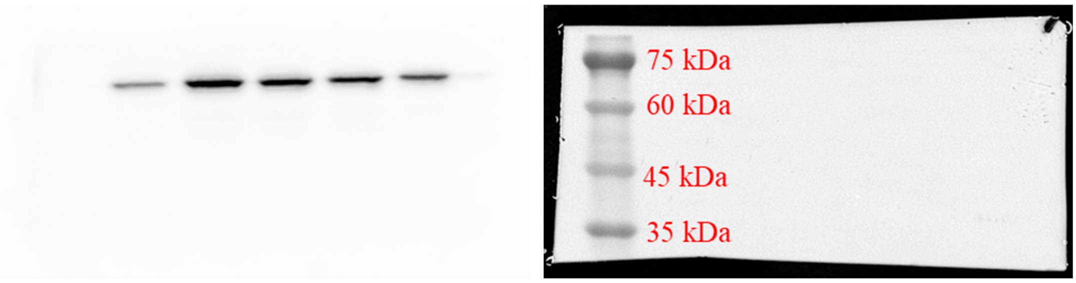

Western blotting analysis for p-p65 (cell sample 1).

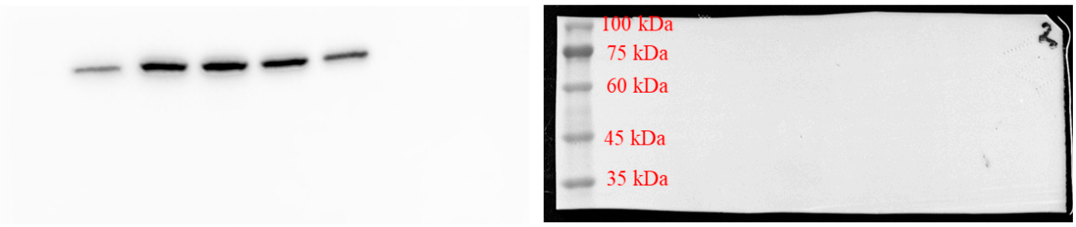

Western blotting analysis for p-p65 (cell sample 2).

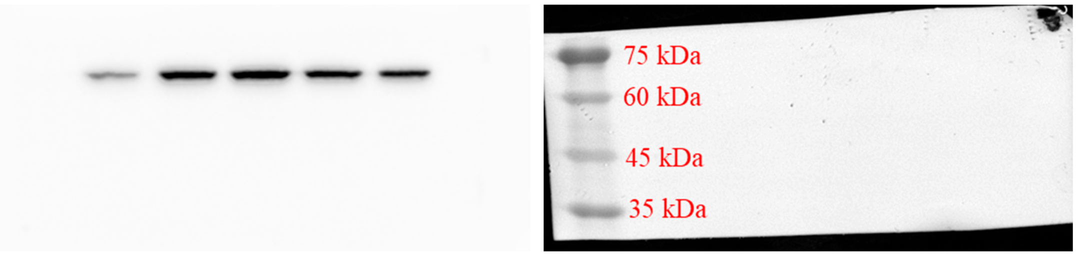

Western blotting analysis for p-p65 (cell sample 3).

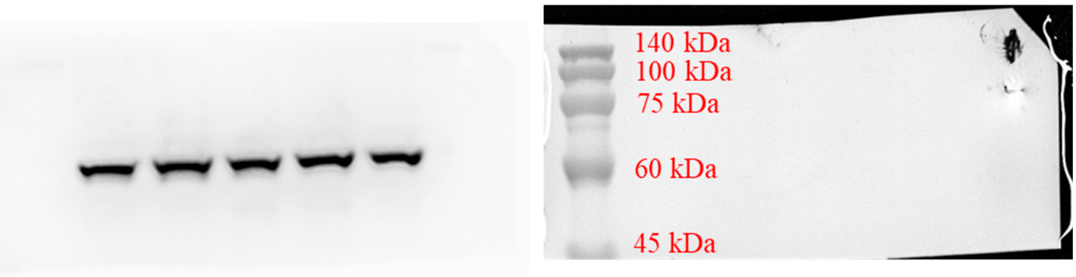

Western blotting analysis for p65 (cell sample 1).

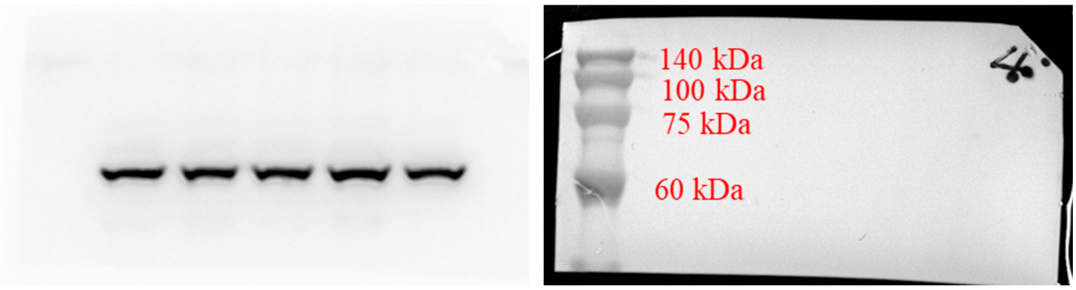

Western blotting analysis for p65 (cell sample 2).

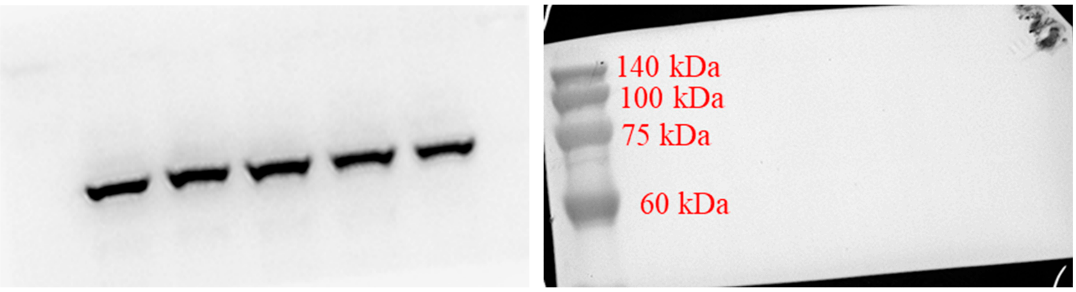

Western blotting analysis for p65 (cell sample 3).

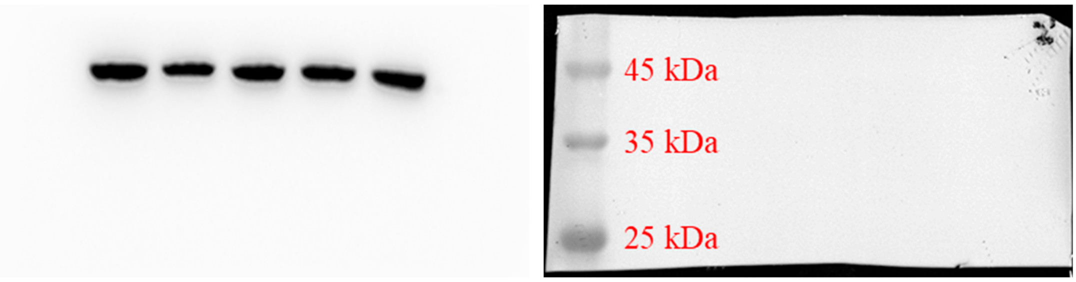

Western blotting analysis for β-actin (cell sample 1).

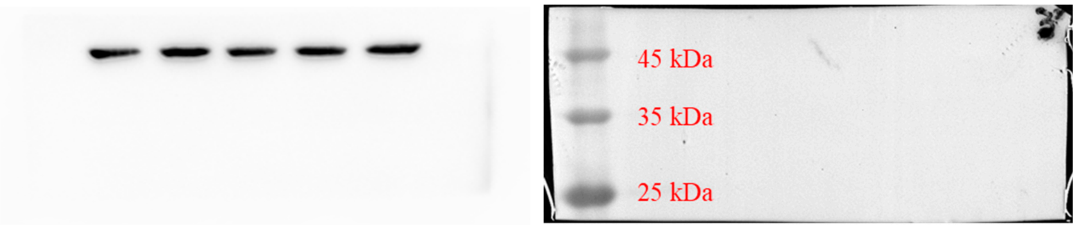

Western blotting analysis for β-actin (cell sample 2).

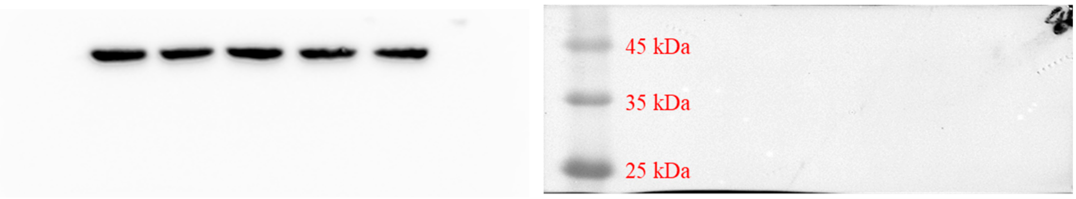

Western blotting analysis for β-actin (cell sample 3).

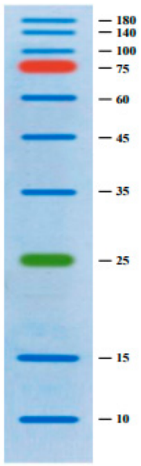

12% SDS-PAGE (Tris-Gly)

Images with prestained protein ladder
