# Supplementary material for: Kaempferol Mitigates Pseudomonas aeruginosa-Induced Acute Lung Inflammation Through Suppressing GSK3β/JNK/c-Jun Signaling Pathway and NF-κB Activation
Source: Pharmaceuticals (Basel). 2025 Feb 25;18(3):322. doi: 10.3390/ph18030322 (PMC11944347; doi:10.3390/ph18030322)
Supplement: Supplementary file 1 [file pharmaceuticals-18-00322-s001.zip › Supplementary data.pdf]

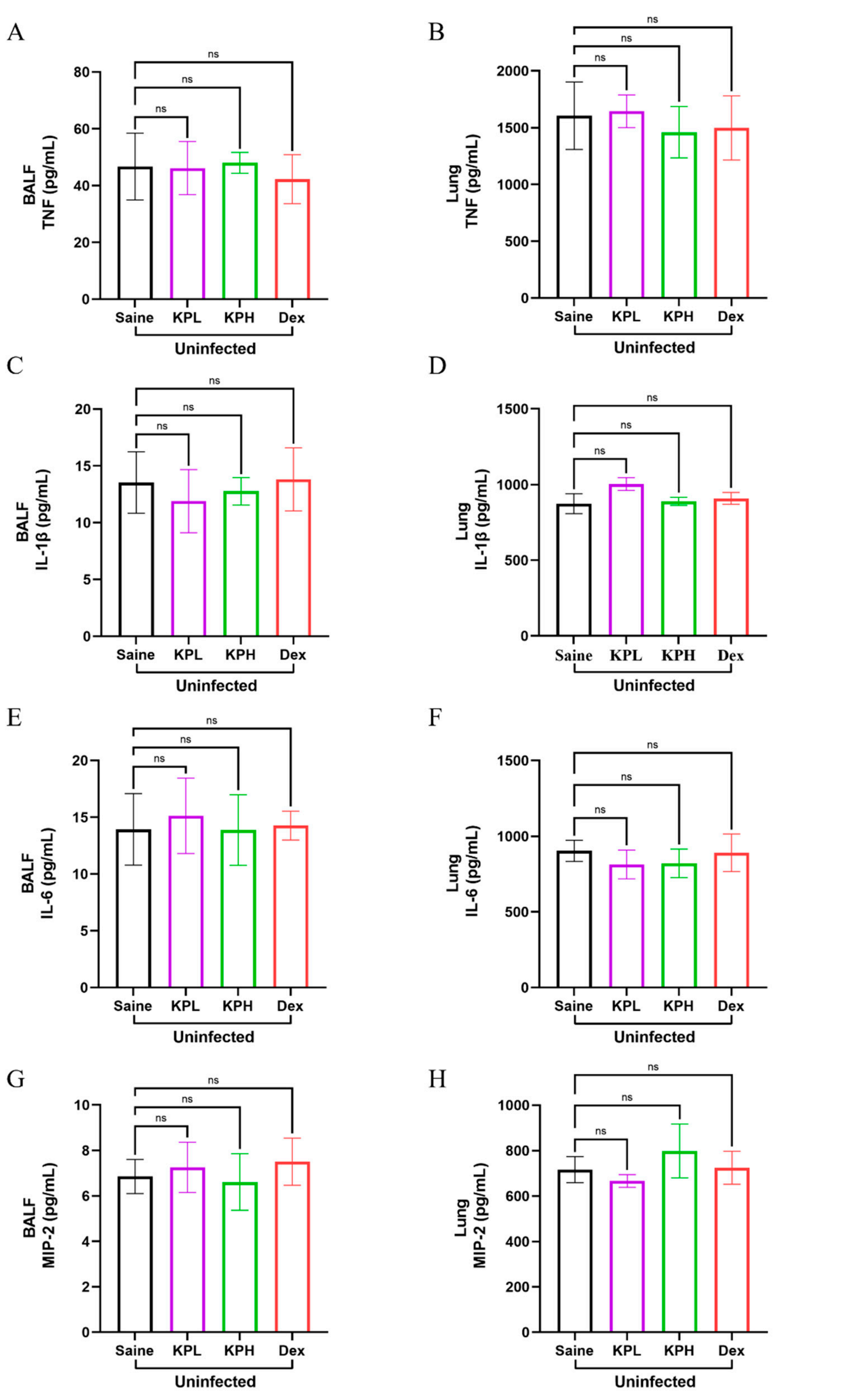


**Figure S1.** KP treatment does not affect the proinflammatory cytokine production in the BALF and lung tissue of uninfected mice. The mice of Saline, KPL, KPH and Dex groups were received a mock infection with saline through intranasal infection for 24 h. The levels of TNF (**A**, **B**), IL-1β (**C**, **D**), IL-6 (**E**, **F**) and MIP-2 (**G**, **H**) in the BALF and lung tissues of the mice were determined by ELISA (data are expressed as mean ± SEM; n = 8; ns: non-significant).

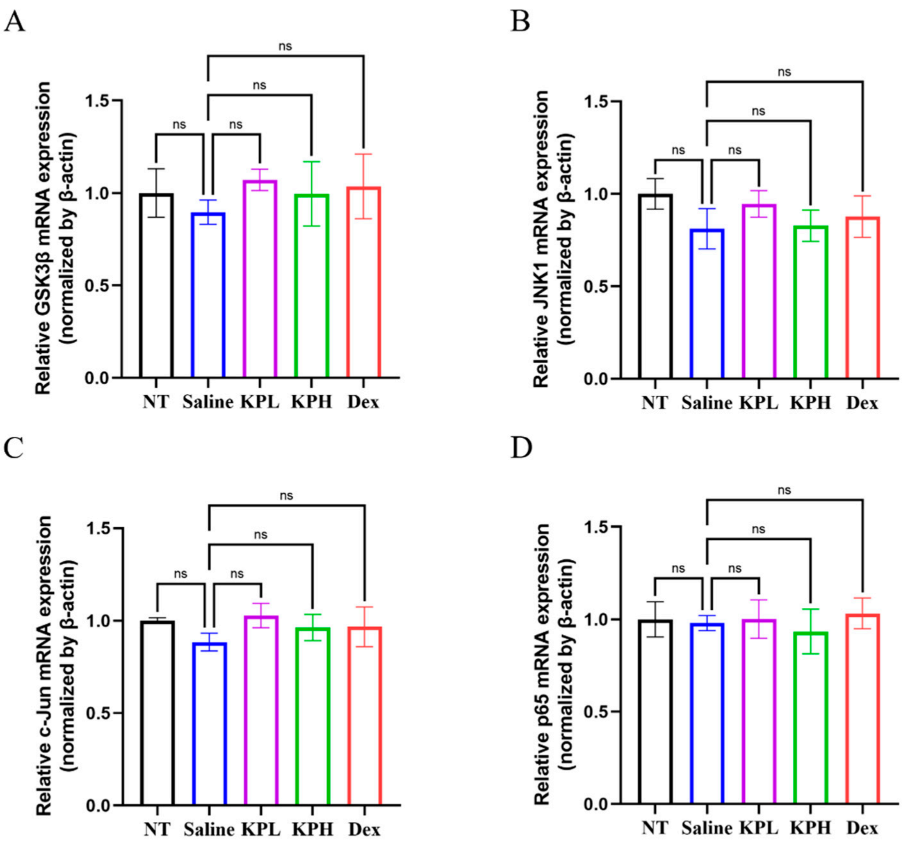


**Figure S2.** KP has no significant effect on mRNA transcription of GSK3β, JNK1, c-Jun and p65 in the lungs of mice during *P. aeruginosa* pulmonary infection. Relative mRNA expression of GSK3β (**A**), JNK1 (**B**), c-Jun (**C**) and p65 (**D**) in the lung tissues of the mice from NT, Saline, KPL, KPH and Dex groups was examined by RT-qPCR (data are expressed as mean ± SEM; n = 3; ns: non-significant). β-actin served as the endogenous control.

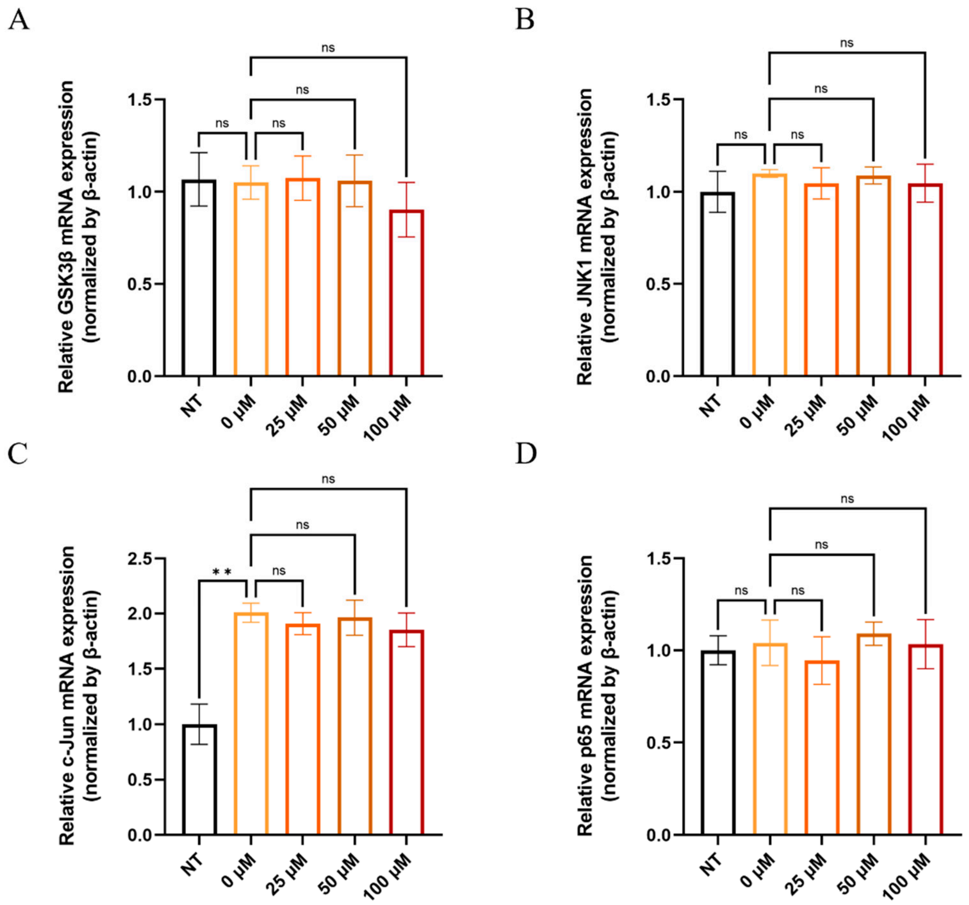


**Figure S3**. KP has no effect on gene expression of GSK3β, JNK1, c-Jun and p65 in macrophages during *P. aeruginosa* infection. BMDMs were pretreated with KP (0, 25, 50 and 100 μM) for 1 h, and then infected with PAO1 or mock-infected for 1 h. Relative mRNA expression of GSK3β (**A**), JNK1 (**B**), c-Jun (**C**) and p65 (**D**) in BMDMs was examined by RT-qPCR (data are expressed as mean ± SEM; n = 3; **p < 0.01, ns: non-significant). β-actin served as the endogenous control.
